# Exploring Metabolic Signatures of Ex Vivo Tumor Tissue Cultures for Prediction of Chemosensitivity in Ovarian Cancer

**DOI:** 10.3390/cancers14184460

**Published:** 2022-09-14

**Authors:** Rita Mendes, Gonçalo Graça, Fernanda Silva, Ana C. L. Guerreiro, Patrícia Gomes-Alves, Jacinta Serpa, Erwin R. Boghaert, Paula M. Alves, Ana Félix, Catarina Brito, Inês A. Isidro

**Affiliations:** 1 iBET, Instituto de Biologia Experimental e Tecnológica, Apartado 12, 2781-901 Oeiras, Portugal; 2 Instituto de Tecnologia Química e Biológica António Xavier, Universidade Nova de Lisboa, Av. da República, 2780-157 Oeiras, Portugal; 3 Department of Metabolism, Digestion and Reproduction, Imperial College London, London SW7 2AZ, UK; 4 iNOVA4Health, NOVA Medical School/Faculdade de Ciências Médicas, Universidade NOVA de Lisboa, Campo dos Mártires da Pátria, 130, 1169-056 Lisboa, Portugal; 5 Molecular Pathobiology Unit, Instituto Português de Oncologia de Lisboa Francisco Gentil (IPOLFG), Rua Prof. Lima Basto, 1099-023 Lisboa, Portugal; 6 AbbVie, 1 North Waukegan Road, North Chicago, IL 60064-6098, USA; 7 Pathology Department, Instituto Português de Oncologia de Lisboa Francisco Gentil (IPOLFG), Rua Prof. Lima Basto, 1099-023 Lisboa, Portugal

**Keywords:** ovarian carcinoma, ex vivo models, tumor microenvironment, metabolomics, drug response, chemoresistance, biomarkers

## Abstract

**Simple Summary:**

Women diagnosed with ovarian cancer have 5-year survival rates below 45%. Prediction of patient’s outcome and the onset of drug resistance are still major challenges. The patient’s drug response is influenced by the environment that surrounds the tumor cells. We previously showed that patient-derived tumor tissue can be kept in the lab, alive and retaining aspects of that environment. In this study, we exposed tumor tissue derived from ovarian cancer patients to the chemotherapy patients receive and identified metabolites released by the tumor tissue after treatment (metabolic footprint). Using machine learning, we uncovered metabolic signatures that discriminate tumor tissues with higher vs. lower drug sensitivity. We propose potential biomarkers involved in the production of specific building blocks of cells and energy generation processes. Overall, we established a platform to explore metabolic features of the complex environment of each patient’s tumor that can underpin the discovery of biomarkers of drug response.

**Abstract:**

Predicting patient response to treatment and the onset of chemoresistance are still major challenges in oncology. Chemoresistance is deeply influenced by the complex cellular interactions occurring within the tumor microenvironment (TME), including metabolic crosstalk. We have previously shown that ex vivo tumor tissue cultures derived from ovarian carcinoma (OvC) resections retain the TME components for at least four weeks of culture and implemented assays for assessment of drug response. Here, we explored ex vivo patient-derived tumor tissue cultures to uncover metabolic signatures of chemosensitivity and/or resistance. Tissue cultures derived from nine OvC cases were challenged with carboplatin and paclitaxel, the standard-of-care chemotherapeutics, and the metabolic footprints were characterized by LC-MS. Partial least-squares discriminant analysis (PLS-DA) revealed metabolic signatures that discriminated high-responder from low-responder tissue cultures to ex vivo drug exposure. As a proof-of-concept, a set of potential metabolic biomarkers of drug response was identified based on the receiver operating characteristics (ROC) curve, comprising amino acids, fatty acids, pyrimidine, glutathione, and TCA cycle pathways. Overall, this work establishes an analytical and computational platform to explore metabolic features of the TME associated with response to treatment, which can leverage the discovery of biomarkers of drug response and resistance in OvC.

## 1. Introduction

Diagnosing chemoresistance and predicting the patient’s outcome is a major challenge in the clinical management of ovarian carcinoma (OvC) [1]. Most affected women are diagnosed at an advanced stage, resulting in 5-year survival rates below 45% [1,2,3]. Among the different histological types of OvC, high-grade serous carcinomas (HGSC) represent the most common type. The standard-of-care (SOC) consists of surgery followed by platinum and taxane-based combination chemotherapy. Both drugs are usually administered every 3 weeks in a total of 6 cycles [4]. Although HGSC patients initially respond to platinum-based chemotherapy, more than 80% experience therapy resistance and tumor recurrence at some stage [5].

Tumor progression and response to treatment are influenced by the complex cell-cell, cell-extracellular matrix (ECM) and cell-soluble factor interactions (including metabolites) that compose the tumor microenvironment (TME) [6,7,8]. In addition, altered cellular metabolism is a hallmark of cancer, and sensitivity to treatment has been correlated with such alterations [9,10,11,12]. Importantly, tumor cell-extrinsic factors and stressful microenvironmental conditions, such as hypoxia and drug challenges, respectively, are increasingly recognized as modulators of the metabolic phenotype of cancer cells, as demonstrated for OvC [13,14,15]. Moreover, metabolic reprogramming of cancer cells also shapes stromal cells’ metabolism [16]. In the specific context of OvC, the so-called Warburg effect (aerobic glycolysis) occurs in cancer-associated fibroblasts (CAFs), a major component of the TME. Consequently, CAFs secrete metabolites, such as amino acids, fatty acids, and lactate, which fuel the surrounding tumor cells. This metabolic symbiosis between CAFs and cancer cells is often called the reverse Warburg effect [17,18,19]. The stromal compartment also plays a significant role in drug response. For instance, CAFs produce glutathione (GSH) that binds to active drugs, thus diminishing their accumulation in cancer cells and contributing to chemoresistance [20]. Importantly, OvC cells present themselves a metabolic reliance on thiol metabolism, and the capacity of GSH turnover is a key feature in sustaining chemoresistance [21].

The molecular and cellular heterogeneity of the TME further complicates the establishment of predictive models of patient response from indirect data, such as blood-based liquid biopsies, demanding especially large patient cohorts due to the low signal-to-noise ratio from the specimen background [22,23]. Patient-derived tumor tissue culture systems retain the architecture and the cellular and non-cellular interactions of the tumor cells and the surrounding TME [8]. Additionally, the use of such cultures to assess drug response and resistance is of utmost interest since several TME traits, important in tumor progression and drug efficacy, are intrinsically present [24]. Therefore, metabolic alterations and drug responses in these complex models are more likely to mimic the in vivo situation [25,26] and consequently potentially more accurate and clinically predictive [8,27].

Herein, we took advantage of an OvC ex vivo model developed by our team. We previously showed that patient-derived OvC tissue can be cultured as explants (OvC-PDE) that sustain the tumor architecture and the TME cellular components for several weeks and are suitable for cyclic drug exposure assays using LDH release as a readout [28,29]. Here, we hypothesized that the modulation of the exometabolome (extracellular metabolome) of the model, i.e., its metabolic footprint, in response to the SOC chemotherapeutics may allow uncovering metabolic signatures of chemosensitivity and resistance. Overall, we established a platform to probe metabolic features of the TME associated with drug efficacy. We present a proof-of-concept of the power of this platform to support the discovery of biomarkers of drug response and resistance and metabolism-targeting drug discovery. Employing supervised learning algorithms, we uncovered metabolic signatures associated with chemosensitivity ex vivo and identified a group of potential biomarkers of drug response that included amino acids, fatty acids, pyrimidine, glutathione, and TCA cycle metabolic pathways.

## 2. Materials and Methods

### 2.1. Study Sample and Histopathological Analysis

Consent was obtained for the use of fresh, surgically removed tumors from nine patients with OvC who underwent surgery at Instituto Português de Oncologia de Lisboa Francisco Gentil (IPOLFG) from 2018 to 2020. Tumor samples (Table 1) were also processed for hemotoxylin and eosin (H&E) staining for epithelium and stroma quantification as previously described [28].

### 2.2. Patient-Derived Explant Culture

Tumor specimens were transported in Dulbecco’s Modified Eagle’s Medium (DMEM, Gibco) supplemented with 10% (*v/v*) Fetal Bovine Serum (Gibco) and 1% (*v/v*) PenStrep (Gibco), from the surgery room to the laboratory and processed under 4 h. Samples were weighed and then mechanically dissociated into fragments of approximately 1 mm^3^, as previously described by our team [28]. These OvC patient-derived explants (OvC-PDE) were cultured in DMEM as described above (culture medium), at 37 °C, 5% CO_2_ in air, in 12 well-plates, at a concentration of 5 PDE/mL, under orbital agitation at 100 rpm, as previously described [29]. The OvC-PDEs were maintained for 21 days in culture and the medium was exchanged every 7 days, as described in [29].

### 2.3. Drug Challenge

The OvC-PDE cultures derived from nine distinct tumor samples were challenged weekly with SOC chemotherapeutics, namely the drug combination of carboplatin (Fresenius Kabi), at a concentration of 25 mg/mL, and paclitaxel (Fresenius Kabi), at a concentration of 10 mg/mL, as previously described by our team [28,29]. The drugs were also administered as single agents, at the same concentrations, and PBS without drugs was added in untreated control cultures. Each condition (untreated control and drug-exposed), for each Ovc-PDE culture, was performed in triplicate. Blank controls were also plated in triplicate: culture medium or culture medium plus drugs, without OvC-PDEs (blank), to assess evaporation and metabolite degradation. To evaluate drug-induced cell death after each cycle of therapy (days 14 and 21), we employed a non-destructive assay, the lactate dehydrogenase (LDH) release assay (Thermo Fisher Scientific, Waltham, MA, USA), performed as we previously described [29].

### 2.4. Conditioned Media Collection and Storage

The conditioned medium (CM) from all conditions (drug-exposed and untreated controls) was collected at days 7, 14 and 21 of culture, as well as the medium from the blank controls. Right after medium collection, samples were centrifuged at 1000× *g* for 5 min, at 4 °C and the supernatant was stored at −80 °C until further metabolomics analysis.

### 2.5. Untargeted Metabolomics

#### 2.5.1. Sample Preparation

Two OvC-PDE cultures were employed in the untargeted metabolic footprinting study: OvC5 and OvC8. A total of 48 CM samples were processed and analyzed: the CMs from days 14 and 21 (untreated controls and exposed to carboplatin, paclitaxel, or the drug combination), all in triplicates. All frozen samples were thawed on ice. For protein precipitation, 300 μL of cold (4 °C) methanol were added to 100 μL of sample and kept at −20 °C, overnight. Samples were vortex-mixed for 10 s and centrifuged at 14,000× *g* for 10 min, at 4 °C. The supernatant was collected and split into two fractions; one fraction was used for direct injection (1 µL) into an amide column. The other fraction was dried using a SpeedVac (Thermo Scientific) and re-suspended in 100 μL of water with 0.1% of formic acid before injection (1 µL) into a C18 column. All samples were analyzed in the same LC-MS run, with quality control (QC) samples to verify the stability of the retention times, peak shapes, and peak areas during the run. The QCs consisted of pooled CM and blank samples and were analyzed every 10 sample injections. Technical LC-MS injection triplicates were acquired for all samples, making up a total of 144 CM measurements.

#### 2.5.2. Liquid Chromatography-Mass Spectrometry

Liquid chromatography-mass spectrometry (LC-MS) analysis was carried out using an UltiMate 3000 UHPLC (Thermo Scientific) system fitted with a Waters XBridge C18 column (2.1 × 150 mm, 3.5 µm particle size, P/N 186003023) for reversed-phase liquid chromatography (RPLC) or with a Waters Acquity UPLC BEH Amide column (2.1 × 150 mm, 2.5 µm particle size, P/N 186003023) for hydrophilic interaction liquid chromatography (HILIC), coupled to a Q Exactive Focus Hybrid Quadrupole-Orbitrap Mass Spectrometer (Thermo Scientific) with an electrospray ionization (ESI) source.

For the RPLC column, water with 0.1% formic acid (mobile phase A) and acetonitrile with 0.1% formic acid (mobile phase B) was used on the gradient elution, using the following program: 0–1 min 1% B; 1–13 min 1–99% B; 13–15 min 99% B; 15–16 min 99–1% B; 16–20 min 1% B. The flow rate was constant at 0.4 mL/min and the temperature was maintained at 30 °C. For the separation carried out in the HILIC column, ammonium acetate at 5 mM (pH 4, adjusted with acetic acid) (mobile phase A) and acetonitrile (mobile phase B) were used on the gradient elution, using the following program: 0–0.1 min 90% B; 0.1–6 min 90–50% B; 6–7 min 50–40% B; 7–9 min 40% B; 9–10 min 40–90% B; 10–20 min 90% B. The flow rate was constant at 0.35 mL/min. The temperature was maintained at 40 °C.

The ESI source was operated in separate runs in both positive and negative ionization modes, with a spray voltage of 3.8 and 3 kV, respectively. The capillary and auxiliary gas heater temperatures were set to 320 °C. The sheath gas and auxiliary gas flow rates were 60 and 20 a.u., respectively. Spectra were acquired in full-MS scan mode (scan range from 75–1125 *m*/*z*) with a resolution of 70,000 (full width at half maximum (FWHM) at 200 *m*/*z*), 1 × 10^6^ automatic gain control (AGC), and internal calibration with lock mass (112.98550 *m*/*z*). A data-dependent method (FullMS-ddMS2) was used to facilitate compound identification. The three most intense ions were subjected to higher-energy collisional dissociation (HCD). A stepped normalized collision energy (NCE) was applied (20, 40 and 60). MS/MS spectra were acquired at 17,500 resolution (FWHM at 200 *m*/*z*) and with automated gain control (AGC) of 1 × 10^5^. The maximum injection time was set to 100 ms and the dynamic exclusion was 6 s.

#### 2.5.3. Data Processing

ProteoWizard was used to convert raw MS data files to the mzML format. These files were then imported into the R environment (version 4.0.3) and R package “XCMS” (Bioconductor version 3.12 [30]) was used for data processing. Four datasets were processed independently, corresponding to HILIC and RPLC columns in both positive (+) and negative (−) ionization modes.

Parameter settings for XCMS processing of data acquired by each column and each mode were as follows: xcmsSet was used to extract the ion chromatograms, followed by peak-picking and grouping, in which each peak was grouped across all samples. A LOESS (nonlinear) regression was used for retention-time correction, followed by grouping. The relative quantification of metabolite features was based on the extracted ion chromatogram (EIC) peak areas. The generated data matrix consisted of the mass-to-charge ratio (*m*/*z*) value, retention time (RT), and peak intensity.

#### 2.5.4. Data Transformation and Normalization

Peak intensity normalization was performed across samples and features in all datasets. Samples were normalized by the median intensity, while log transformation and unit-variance scaling were applied across features, so that peak intensity data acquired a Gaussian-like distribution. The normalization strategy was evaluated by checking the symmetry and range of the boxplots of feature intensities and using principal component analysis (PCA) to check the clustering of QC data points. Data pre-processing and subsequent analysis steps were carried out using the MetaboAnalyst 4.0 Web Server [31].

#### 2.5.5. Analytical Validation and Outlier Detection

The quality of the analytical system performance was evaluated using PCA, which was also used to detect possible outliers. The score matrix from PCA was assessed for PC1 and PC2. Outliers were evaluated according to the sample principal component (PC) score by visual inspection of the maximum variance of the main data explained by the PC1 and PC2. In the HILIC(+) dataset, three data points (three replicates of injection of one CM sample, corresponding to one of the three independent cultures from OvC5 in the untreated condition at day 14) were identified as outliers. In the HILIC(−) dataset, no data points were identified as outliers. In the RPLC(+) dataset, three data points (three replicates of injection of one CM sample, corresponding to one of the three independent cultures from OvC8 in the untreated condition at day 14) were identified as outliers. In the RPLC(−) dataset, one data point (one of the three replicates of injection of one CM sample from OvC8 in the untreated condition at day 14) was identified as an outlier. QC samples were included on a PCA plot to assess the stability of the analytical system. The analytical runs are considered valid if QCs data points are well clustered in the PCA and stable in PC1 throughout the injection run.

#### 2.5.6. Statistical Analysis

The normalized data were further used for multivariate data analysis. Principal component analysis was used to reduce the dimensionality and to identify clusters among the different treatment conditions. Partial least-squares discriminant analysis (PLS-DA) was then applied to find the discriminant features among treatment conditions. To assess the optimal number of components to build the PLS-DA model 5-fold cross-validation was used. The performance of the model was evaluated by the accuracy (significance of class discrimination), R^2^ and Q^2^ parameters, and by a permutation test (100 permutations). Variable importance in projection (VIP) score, given by the PLS loadings, was used to rank the most relevant features for group discrimination. We focused on the VIP scores from the first component since it always explained a large part of the total variance. For good performance models (accuracy, R^2^ and Q^2^ > 0.9), the top 25 discriminant features were selected according to the VIP scores for each data set. Thus, 75 features were retained for metabolite identification.

#### 2.5.7. Metabolite Identification

Metabolite identification was performed for 75 discriminant features resulting from the VIP score analysis. First, isotopic patterns and adduct ions of tentative candidates were searched against the Human Metabolome Database (HMDB) [32]. The list of tentative candidates was reduced by LogP evaluation according to the column. Briefly, tentative candidates with LogP > 0 were excluded in HILIC datasets and LogP < 0 in RPLC datasets. Finally, MS/MS spectra were compared to experimental MS/MS spectra from pure compounds deposited in HMDB [32], METLIN [33] and GNPS [34] databases. For cases where MS/MS spectra were not available in those databases, fragmentation patterns were manually interpreted for metabolite annotation. Putative identifications were obtained and annotated according to the proposed workflow for metabolite identification confidence by Schrimpe–Rutledge et al. [35].

### 2.6. Semi-Targeted Metabolomics

#### 2.6.1. Semi-Polar Metabolite Extraction, Identification, and Relative Quantification

Technical duplicates of all PDE cultures (OvC1–9) at days 14 and 21 of culture (untreated controls and drug challenge conditions) were analyzed as follows by MS-Omics (Copenhagen, Denmark). The analysis was performed using a Thermo Scientific Vanquish LC coupled to Thermo Q Exactive HF MS. An electrospray ionization interface was used as the ionization source. Analysis was performed in negative and positive ionization modes. The UPLC was carried out using a slightly modified version of the protocol described by Catalin et al. [36]. Peak areas were extracted using Compound Discoverer 3.1 (Thermo Scientific). Identification of compounds was performed at four levels; Level 1: identification by retention times (compared against in-house authentic standards), accurate mass (with an accepted deviation of 3 ppm), and MS/MS spectra, Level 2a: identification by retention times (compared against in-house authentic standards), accurate mass (with an accepted deviation of 3 ppm). Level 2b: identification by accurate mass (with an accepted deviation of 3 ppm), and MS/MS spectra, Level 3: identification by accurate mass alone (with an accepted deviation of 3 ppm). Out of 1382 metabolites, 51 were identified based on the accurate mass, MS/MS spectra and known retention time obtained from standards (confidence level 1) and 36 based on accurate mass and known retention time obtained from standards (level 2a). In addition, 38 compounds were identified based on the accurate mass and MS/MS spectra from an external library (level 2b), 85 based on library searches using the accurate mass and elemental composition alone (level 3), and 1172 compounds remain unidentified.

#### 2.6.2. Statistical Analysis

Data normalization was carried out, as for the untargeted dataset, by applying the median, log and unit variance normalization using the MetaboAnalyst 5.0 Web Server [37]. The PCA was built using GraphPad Prism software (version 9) and MetaboAnalyst 5.0 Web Server [37]. Supervised models, namely PLS-DA, sparse(s)PLS-DA and random forest (RF) analysis were carried out using the MetaboAnalyst 5.0 Web Server. The performance of the PLS-DA was evaluated by the accuracy (significance of class discrimination), R^2^ and Q^2^ parameters; sPLS-DA by the error rate by number of components; and RF by the out-of-bag (OOB) error. We used VIP score analysis to select the top 25 discriminant features for PLS-DA model, loading scores were used for sPLS-DA and mean decrease accuracy was used for RF. Biomarker analysis was accomplished using the MetaboAnalyst 5.0 Web Server. Multivariate receiver operating characteristic (ROC) curve exploration was performed using PLS-DA algorithm, with 2 latent variables (LV). ROC curves are generated by Monte Carlo cross-validation (MCCV) using balanced subsampling. In each MCCV, 2/3 of the samples are used to evaluate feature importance, and the remaining 1/3 are used to validate the models created with the first step. The top-ranking features (up to 100) in terms of importance are used to build the biomarker classification models the performance and confidence intervals (CI) of each model are calculated. Principal component regression (PCR) was carried out in GraphPad Prism software (version 9).

#### 2.6.3. Metabolic Pathway Analysis

Control and combination-treated normalized peak intensity datasets categorically classified into high-responders (HR) and low responders (LR) were used for pathway analysis. First, 85 out of 87 compound names were successfully converted to identifiers used in HMDB [32]. Homo sapiens (KEGG) was selected as the pathway library, the global test as the algorithm for the pathway enrichment analysis and the node importance measure for topological analysis selected was relative betweenness centrality. The visualization method selected was a scatter plot (testing significant features). To find additional important pathways, the top common (across models) discriminant metabolites for HR vs. LR classification obtained after supervised analysis of the reduced datasets of untreated controls and drug combination-treated conditions were searched. For that, the Small Molecule Pathway Database (SMPDB) was used with 99 metabolite sets based on normal human metabolic pathways and in KEGG. All metabolic pathway analyses were carried out using MetaboAnalyst 5.0 Web Server.

## 3. Results

### 3.1. Study Design, Characterization of the Patient Cohort, and Chemotherapy Assessment in Patient-Derived Explant Cultures

**Figure 1:**
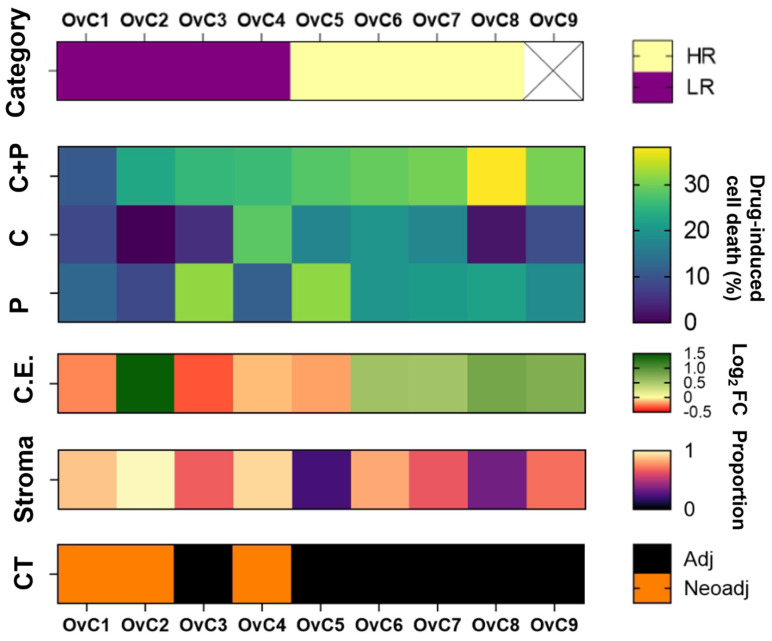
Sample-specific response to combination (carboplatin + paclitaxel) and single drugs cyclic drug exposure in serous ovarian carcinoma patient-derived ex vivo cultures and clinical information of the patient cohort. Ovarian carcinoma (OvC) patient-derived explants (PDE) cultures are categorized into high-responders (HR) and low-responders (LR) according to the ex vivo drug-induced cell death upon the first drug combination cycle, using the median as cut-off (OvC9 was excluded as it was the only low-grade serous carcinoma PDE). Patient sample-specific differences in drug-induced cell death, combination effect (C.E.), stroma proportion and clinical chemotherapy (CT) regimen are captured in the heatmap, (C: carboplatin; P: paclitaxel).

The OvC patients usually undergo surgery followed by adjuvant chemotherapy, consisting of a combination of carboplatin and paclitaxel. Surgically resected tumor samples from nine patients diagnosed with serous epithelial OvC were enrolled in this study (Table 1), which included one low-grade serous carcinoma (LGSC) and eight HGSC cases. After surgery, six of the patients received six cycles of adjuvant chemotherapy. The other three patients underwent three cycles of neoadjuvant chemotherapy and three cycles of adjuvant chemotherapy (Figure 1). The histopathological analysis revealed that seven of the nine tumor samples presented a high stroma (>50%) to epithelium proportion (Figure 1). Clinical and pathological information are reported in Table 1.

**Table 1:** Ovarian Carcinoma patient cohort: clinical data and patient-derived explant (PDE) culture classification.

Case	Clinical Information						PDE Culture
	Age	Diagnosis	FIGO	Chemotherapy	NACT Evaluation	Patient Status (Follow-Up at 10 Months)	Response to Drug Combination
OvC1	62	HGSC	IIIC	Neoadjuvant + adjuvant	CRS1	Alive with disease	LR
OvC2	51	HGSC	IIIC	Neoadjuvant + adjuvant	CRS3	No evidence of cancer disease	LR
OvC3	44	HGSC	IIIC	Adjuvant	-	No evidence of cancer disease	LR
OvC4	62	HGSC	IIIC	Neoadjuvant + adjuvant	CRS2	Alive with disease	LR
OvC5	81	HGSC	IIIC	Adjuvant	-	Alive with disease	HR
OvC6	52	HGSC	IIIC	Adjuvant	-	Alive with disease	HR
OvC7	68	HGSC	IIIC	Adjuvant	-	No evidence of cancer disease	HR
OvC8	77	HGSC	IIIC	Adjuvant	-	Alive with disease	HR
OvC9	85	LGSC	IIIB	Adjuvant	-	Died with cancer disease	-

OvC: ovarian carcinoma; HGSC: high-grade serous carcinoma; LGSC: low-grade serous carcinoma; NACT: neoadjuvant chemotherapy; CRS1: no/minimal response; CRS2: partial response; CRS3: complete/near complete response; HR: high-responder; LR: low-responder.

**Figure 2:**
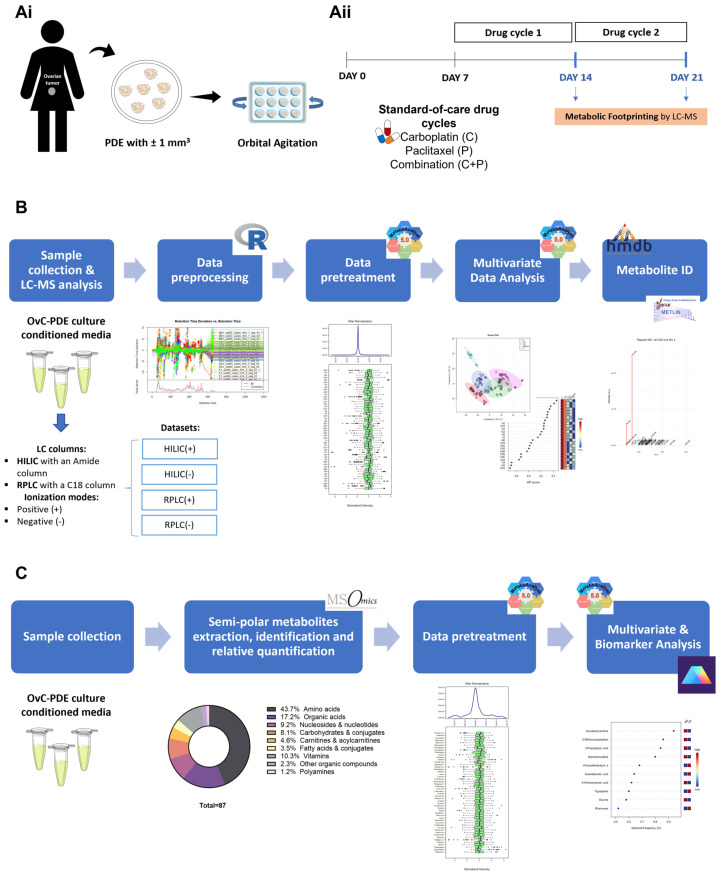
Workflow of the experimental strategy for (**Ai**) patient-derived ex vivo (PDE) culture establishment, (**Aii**) drug assays, approach, and readouts, (**B**) untargeted metabolomic analysis and (**C**) semi-targeted metabolomics. (OvC: ovarian carcinoma; PDE: patient-derived explant; LC-MS: liquid chromatography-mass spectrometry; HILIC: hydrophilic interaction liquid chromatography; RPLC: reversed phase liquid chromatography; ID: identification).

We developed an OvC patient-derived PDE culture (Figure 2(Ai)) [28] in which tumor architecture and cell-type heterogeneity (epithelial and stromal compartments) were preserved for at least one month in culture. This culture strategy can be broadly applied to the culture of different OvC types [28]. Here, we used the downscaled version of this model, as characterized by Cox and Mendes et al. [29] and processed as described in the methods section (Figure 2(Ai)).

The OvC-PDE cultures were challenged with the SOC chemotherapeutics, namely the combination of carboplatin and paclitaxel (C+P), or single-agent chemotherapy (Figure 2(Ai)). The PDEs were exposed to the drugs in two consecutive cycles of 1 week and the drug-induced cell death was evaluated after each cycle using the LDH assay. To explore whether metabolic footprinting can be used to evaluate drug response in PDE cultures, we collected the CM after each drug cycle (Figure 2(Aii)).

To analyze the effect of the combination therapy, we compared the efficacy of C+P with the most efficacious single agent, henceforth referred to as the highest single agent (HSA) [38] (Figure S1A,C). For most cases, the HSA was paclitaxel, except for OvC4 and OvC6 in which it was carboplatin (Figure S1A). We observed a negative combination effect (log_2_ fold-change (log_2_ FC) < 0), meaning the combination was less efficacious than the HSA, in four out of nine (44%) OvC-PDE cases. In the remaining five (56%) cases, we observed a positive combination effect (log_2_FC > 0), meaning the combination therapy was more efficacious than the HSA (Figure S1C). Interestingly, cases in which patients received neoadjuvant chemotherapy (OvC1, OvC2 and OvC4), clinical evaluation matched with log_2_ FC evaluation, where log_2_ FC = −0.24 was observed for OvC1 (no/minimal response), log_2_ FC = −0.13 was observed for OvC4 (partial response) and log_2_ FC = 1.45 was observed for OvC2 (complete/near-complete response) (Figure 1 and Table 1). Among the eight HGSC PDE cultures, four cases (OvC1–4) were considered low-responders (LR) and the other four (OvC5–8) high-responders (HR), using as cut-off the median drug-induced cell death upon the first drug combination cycle (Figure 1 and Table 1).

### 3.2. Untargeted Metabolic Footprints Distinguish between Treatment Groups—A Proof-of-Concept

To verify whether the analysis of the exometabolome, i.e., metabolic footprinting can detect distinct responses depending on the PDE treatment condition and assess the robustness of the culture sampling and analytical platform to capture these different profiles, we applied an untargeted liquid chromatography-mass spectrometry (LC-MS)-based methodology to two HGSC-PDE cultures (OvC5 and OvC8). We collected the conditioned medium (CM) after each drug cycle (days 14 and 21 of culture, corresponding to 7 and 14 days of drug exposure, respectively, Figure 2(Aii) and Figure 2B).

**Figure 3:**
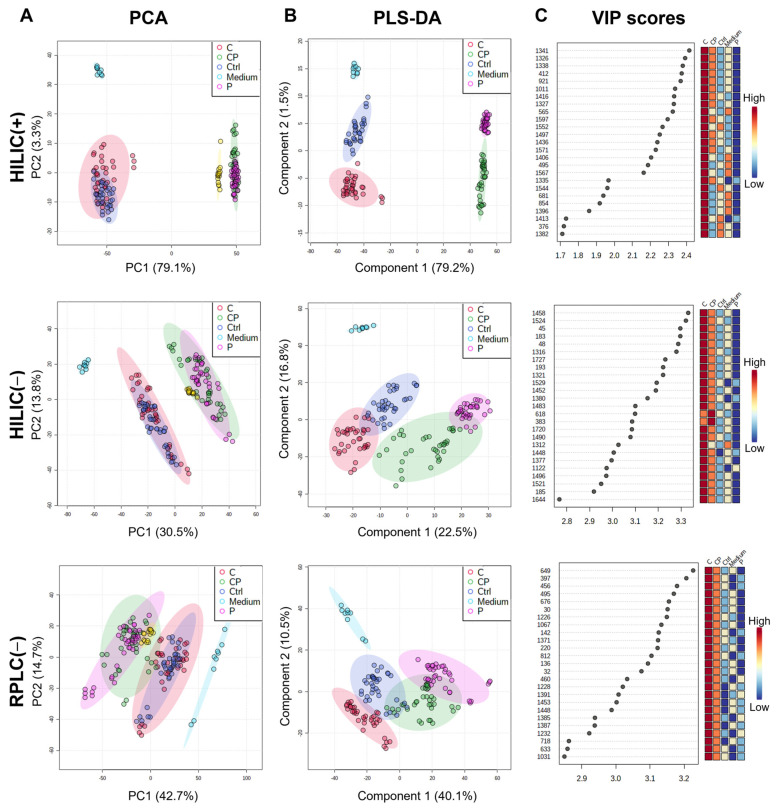
Untargeted metabolic footprints can distinguish between treatment groups in two high-grade serous ovarian carcinoma (OvC) patient-derived explant (PDE) cultures. Multivariate data analysis performed using: (**A**) principal component analysis (PCA), (**B**) partial least-squares discriminant analysis (PLS-DA) for subsequent identification of corresponding important features identified by (**C**) variable importance in projection (VIP) score in PLS-DA (colored boxes on the right indicate the relative concentrations of the corresponding metabolite in each condition). The LC-MS-based metabolic footprinting performed using HILIC and RPLC columns, in positive (+) and negative (−) ionization modes; N = 2 OvC cases (OvC5 and OvC8), with 3 culture replicates per condition, and 3 LC-MS injections each, for a total of 144 PDE-conditioned media data points shown (**C**: carboplatin, P: paclitaxel; Ctrl: untreated control; Medium: culture media blanks; HILIC: hydrophilic interaction liquid chromatography; RPLC: reversed-phase liquid chromatography; (+): positive mode; (−): negative mode; PC: principal component).

We used two columns (HILIC and RPLC) and two ionization modes (positive and negative) to broaden the metabolite coverage, making up four datasets. A total of 3153 features (retention time, *m*/*z* pairs) were extracted in the HILIC(+) dataset; 2036 in HILIC(−); 3334 in RPLC(+); and 1933 in RPLC(−) (Supplementary File S2). The retention time drift correction and LC-MS EICs of the detected features are shown in Figure S2. After data normalization (Figure S3i), QCs cluster together (Figure 3A) and are stable throughout the injection run (Figure S3ii), indicating an adequate performance of the analytical and data processing platform. The RPLC(+) dataset was excluded from further analysis since it did not have consistent QC measurements (Figure S4).

The PCA score plot of metabolic footprints (Figure 3A) revealed different drug response groups in HILIC(+), HILIC(−) and RPLC(−) datasets. The first two principal components (PC) accumulated 82.4% of the total explained variance in the HILIC(+), 44.3% in the HILIC(−), and 57.4% in the RPLC(−) dataset. Group separation is mainly achieved by the first PC, and three major clusters are observed: untreated control grouped with the carboplatin-treated condition; paclitaxel-treated condition grouped with the combination-treated condition; and culture medium blanks. The emergence of treatment clusters in an unsupervised method like PCA supports the existence of characteristic metabolic signatures. Thus, sample discrimination was further analyzed using a supervised approach by PLS-DA.

The PLS-DA models to distinguish among treatment conditions based on the datasets HILIC(+), HILIC(−) and RPLC(−) showed high accuracy, R^2^ and Q^2^ parameters (>0.9) for a 5-fold cross-validation (Figure S5i). The prediction accuracy during training was also assessed by permutation analysis (100 permutations) and, for all models, discrimination was considered significant (*p*-value < 0.01) (Figure S5ii). The PLS-DA (Figure 3B) of metabolic footprints found discriminant features between drug treatment groups for subsequent identification of corresponding metabolites. For each supervised model built from the three initial feature datasets, a panel of the top 25 metabolic features was selected based on the variable importance in projection (VIP) score measured in loading 1 of PLS-DA (Figure 3C).

Metabolite identification was attempted for the 75 top discriminant features, comprising the top 25 from each dataset. First, EICs and the corresponding mass spectra were obtained for each feature. Mass spectral detected adduct ions and elemental formula of possible candidates were searched in the HMDB [32] database. Subsequently, fragmentation patterns obtained from tandem MS experiments (when available) were compared to MS/MS spectra in the HMDB [32], Metlin [33], or GNPS [34] databases. Additionally, metabolites present in the basal medium used to culture OvC-PDEs, namely DMEM, were also searched only by comparing MS/MS spectra with the previously mentioned spectral libraries (Supplementary File S2, Figures S6 and S7).

Some of the top features correspond to the same compound because adduct grouping was not performed beforehand, although these were easily identified through the isotopic pattern analysis. Features were identified as [M + H]^+^ ionic species in positive ionization datasets and as [M − H]^−^ or [M + Cl]^−^ ionic species in negative ionization datasets. To filter the number of candidates obtained in the accurate MS search, logP was considered as described in the methods section. Putative identifications with different levels of annotation confidence were achieved for 33 features, annotated according to Schrimpe–Rutledge et al. [35]. Overall, out of 75 top features, 30 features were identified based on the fragmentation data matched to metabolite MS/MS libraries (confidence level 2), three remain as tentative structures in which precursor *m*/*z* matched to a metabolite database (confidence level 3), and 42 remained unknown (confidence level 5). However, by performing adduct grouping, the 33 identified features were translated into 10 compounds, which belong to amino acids (2), peptides (2), carnitines and acylcarnitines (1), fatty acids and conjugates (2) and vitamins (1) metabolite classes. Carboplatin, one of the drugs used in this study, was also identified in both HILIC datasets ((+) and (−)). In addition, seven metabolites (5 amino acids, 1 vitamin and 1 carbohydrate) known to be present in DMEM culture media were identified (confidence level 2: 5 features, confidence level 3: 2 features) (Supplementary File S2).

### 3.3. Semi-Targeted Exometabolome Analysis Captures Metabolic Heterogeneity between High and Low-Responders

As metabolic footprinting revealed differences in response to drug treatment in PDE cultures, a semi-targeted metabolomics approach was then employed, analyzing the metabolite classes of the most relevant features identified in the untargeted metabolomics experiment, using a semi-polar LC-MS/MS method (Figure 2C). Further data analysis was carried out using metabolites annotated with high confidence based on standards (levels 1 and 2a, see Methods section), i.e., 87 metabolites (Supplementary File S3), mainly belonging to amino acids (44%); organic acids (17%); vitamins (10%); nucleosides and nucleotides (9%); carbohydrates and conjugates (8%); carnitines and acylcarnitines (5%); and fatty acids and conjugates (3%) classes (Figure 2C).

**Figure 4:**
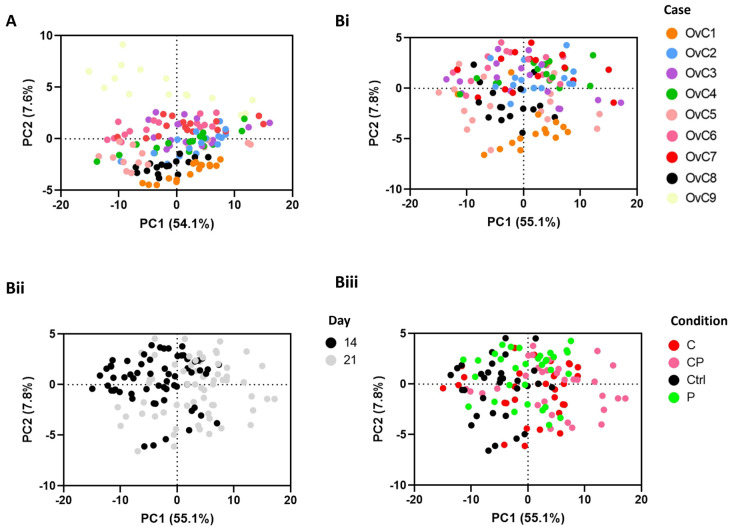
Semi-targeted exometabolome analysis reveals metabolic heterogeneity among serous carcinoma-derived explant cultures. Multivariate data analysis using principal component analysis (PCA) reveals clustering trends by OvC case (**A**) across all samples, including untreated controls (Ctrl) and treated conditions, at days 14 and 21, and (**Bi**) between high-grade serous carcinoma (HGSC)-derived explant cultures (i.e., excluding OvC9). The latter also reveals (**Bii**) a slight trend by timepoint and (**Biii**) no clear separation by treatment group (C: carboplatin, P: paclitaxel, CP: drug combination).

We analyzed CM duplicates of all experimental conditions of each OvC-PDE culture (Figure 2(Aii)). After normalizing across samples and features the QC data points clustered together in PCA (Figure S8). In addition, we identified distinct groups, in which OvC9, derived from an LGSC explant, stood apart from the HGSC-derived cultures (Figure 4A). This separation was visible specifically along PC2, which seems to discriminate by OvC-PDE case, including to some extent within the HGSC cases. Further analysis only considered HGSC OvC-PDE cultures (Figure 4B).

The PCA based on CM samples from HGSC-derived cultures (Figure 4(Bi–Biii)), in which the first two PCs accounted for 62.9% of the total explained variance, revealed also a trend by timepoint (day 14 or 21), while a clear trend by drug treatment condition was not identified (Figure 4(Bii,Biii)). The latter might be a consequence of intrinsic metabolic heterogeneity among PDE cultures derived from different patients.

**Figure 5:**
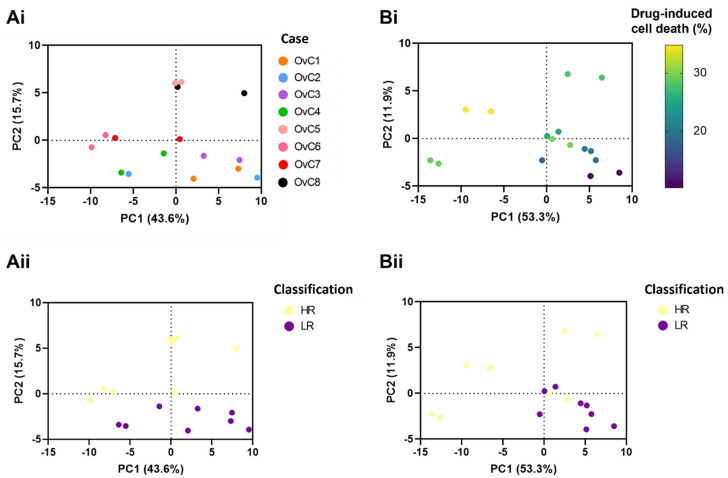
Metabolic footprinting captures metabolic differences between high- and low-responders in untreated controls and combination-treated patient-derived explant cultures. Principal component analysis (PCA) of metabolic footprints of high-grade serous carcinoma (HGSC)-derived explant cultures, at day 14, (**A**) in untreated control conditions predicted different drug response trends by (**Ai**) OvC case and (**Aii**) when categorized by the ex vivo drug response to drug combination. (**B**) In drug combination conditions, PCA revealed drug response trends by (**Bi**) drug-induced cell death after one drug cycle (day 14) and (**Bii**) based on ex vivo drug response categories. (HR: high-responders, LR: low-responders, classified using the drug combination-induced cell death at day 14, with the median as cut-off; PC: principal component).

In fact, by reducing the dataset complexity and visualizing the untreated controls at day 14 (Figure 5(Ai)), two clusters that separate HR (OvC5–8) from LR (OvC1–4) to the drug combination are identified (Figure 5(Aii)). The first two PCs accumulate 59.3% of the total explained variance.

To verify whether the metabolic footprint distinguished the efficacy of the drug combination regimen, we focused on the dataset generated from PDE cultures challenged with the combination of carboplatin and paclitaxel, at day 14. The tendency observed in the drug combination-treated PDE cultures dataset was the same as in the untreated controls dataset. The PCA (the first two PCs accumulated 65.2% of the total explained variance) identified different drug response groups based on the ex vivo drug-induced cell death (Figure 5(Bi,Bii)) and PDE case-specific trends (Figure S11(Ai)). Specifically, in the PCA for the drug combination, a gradient from the right lower quadrant (almost all LR) to the left upper quadrant (HR spread in the other three quadrants) was observed (Figure 5(Bi)).

Although it is not the focus of our study, as single drugs are not administered in the clinical setting, the PCA based on the single drugs dataset, i.e., PDE cultures treated with carboplatin or paclitaxel, also separated HR from LR (Figure S9). Specifically, the PCA score plot for single agents (the first two PCs accumulated 59.2% of the total explained variance) revealed different drug response groups observed by a gradient from the left upper quadrant (LR) to the right lower quadrant (HR) and by HR vs. LR category (Figure S9(Aiii,Aiv)), as well as different treatment groups mostly separated on PC2 (Figure S9(Aii)).

**Figure 6:**
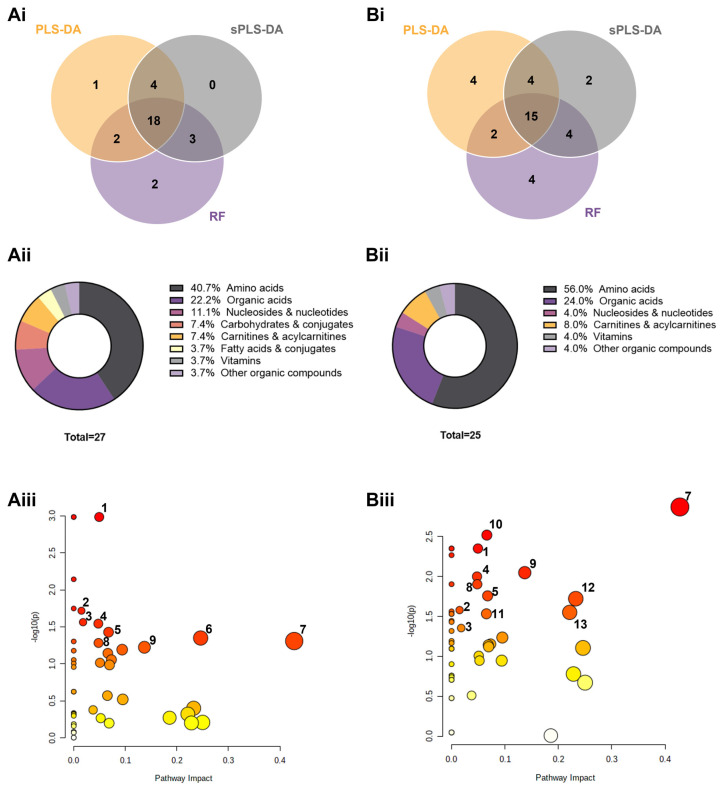
Supervised learning and pathway analysis from metabolic footprinting of patient-derived explant cultures reveals discriminating metabolic features between high- (HR) and low-responders (LR). For (**A**) untreated control and (**B**) drug combination-treated HGSC datasets at day 14, supervised models (PLS-DA: partial least-squares discriminant analysis; sPLS-DA: sparse PLS-DA; RF: random forests) revealed (**i**) consistent top metabolic features discriminating HR from LR and (**ii**) respective metabolite classes. (**iii**) Pathway analysis of HR vs. LR shows significantly altered pathways for fatty acids metabolism (1, 2), amino acids metabolism (3, 4, 6, 7, 8, 9) and pyrimidine metabolism (5) pathways. A list of metabolites and respective classes, as well as altered pathways and respective metabolites, are included in Supplementary File S4, (color scale and symbol size reflect the combination between the *p*-value and the pathway impact, respectively).

To find the metabolites with the most predictive value for discrimination between the HR and LR classes in the untreated control condition, three machine learning algorithms were applied (PLS-DA, sPLS-DA and RF; Figure S10A–C) and the top 25 discriminant features of each model were compared. Out of 30 unique metabolites among the three models, 27 intersected in at least two of the three models (Figure 6(Ai)), which were considered to carry the foremost discriminant power. Amino acids (40.7%), organic acids (22.2%) and nucleosides and nucleotides (11.1%) are among the most relevant metabolites accounting for the differences in metabolic phenotype between HR and LR in the untreated control dataset (Figure 6(Aii)). Once again, the same strategy was applied in the drug combination dataset. Out of 35 unique metabolites among the three models, 25 were common to at least two models (Figure 6(Bi)). The most important metabolites separating the metabolic footprints of HR and LR after the drug challenge were amino acids (56.0%), organic acids (24.0%) and carnitines and acylcarnitines (8.0%) (Figure 6(Bii)).

Additionally, principal component regression (PCR) using the drug combination dataset allowed a fine discrimination (*p*-value = 0.0001) of the drug-induced cell death as a continuous variable (Supplementary File S4). The PCR also showed a correlation (*p*-value = 0.002) between the PDE metabolic footprint and the patient chemotherapy regimen (neoadjuvant only vs. neoadjuvant + adjuvant; Figure S11(Aii) and Supplementary File S4).

Among the 39 top metabolites discriminating HR and LR in both untreated control and drug combination datasets, there were 14 that appeared only in untreated controls and 13 that were common to both conditions. An additional set of 12 metabolites was altered only in the drug combination dataset (Figure S12A). The 27 metabolites identified in the untreated controls were considered metabolic traits intrinsic to the OvC-PDE case, and the 12 metabolites identified only in the drug combination dataset were considered as alterations induced by the drug response. The major differences in metabolic classes were in amino acids, which comprised 66.7% of the top altered features upon the drug challenge (Figure S12B).

Because unsupervised and supervised analyses uncovered differences between HR and LR in both untreated control and drug combination datasets, we used both datasets for metabolic pathway analysis. The metabolic differences between HR and LR based on the untreated condition dataset relied on: (i) fatty acids metabolism, specifically in beta-oxidation of very-long-chain fatty acids and fatty acid biosynthesis; (ii) amino acids metabolism, namely valine, leucine and isoleucine degradation, glycine and serine, arginine and proline, histidine, methionine, and betaine metabolism; and (iii) pyrimidine metabolism (Figure 6(Aiii)). Likewise, we found the following pathways significantly altered between HR and LR in the drug combination condition: (i) amino acids metabolism, namely histidine, beta-alanine, betaine, glycine and serine, methionine, phenylalanine and tyrosine metabolism and valine, leucine and isoleucine degradation; (ii) fatty acids metabolism, specifically in beta-oxidation of very-long-chain fatty acids and fatty acid biosynthesis; (iii) pyrimidine metabolism; and iv) tricarboxylic acid (TCA) cycle and transfer of acetyl groups into mitochondria (Figure 6(Biii)). Furthermore, to expand the understanding of metabolic alterations, we performed pathway analysis of the 27 metabolites intrinsic to the OvC-PDE case (untreated condition), and the 12 metabolites induced by the drug combination, that distinguished HR from LR. We found four additional pathways, including tryptophan (tryptophan and 5-methoxytryptophan), purine (1-methyladenosine), vitamin B6 (pyridoxal), and cysteine and methionine metabolism (cystine) related to the top PDE case-intrinsic metabolites (Supplementary File S4 and Figure S12). For the drug combination-induced metabolites, we found additional alterations in GSH metabolism (pyroglutamic acid) and vitamin B6 metabolism (pyridoxine) (Supplementary File S4).

### 3.4. Metabolic Signatures as Potential Biomarker Panels to Predict Ex Vivo Drug Efficacy

Supervised learning uncovered metabolic signatures that differentiated HR from LR, that were consistent using linear and nonlinear/ensemble algorithms. Thus, potential multi-biomarker panels were explored using multivariate exploratory receiver operating characteristics (ROC) analysis, generated using PLS-DA as a classification algorithm and feature ranking method. This methodology has been widely applied for assessing the discriminant performance for biomarkers [39].

**Figure 7:**
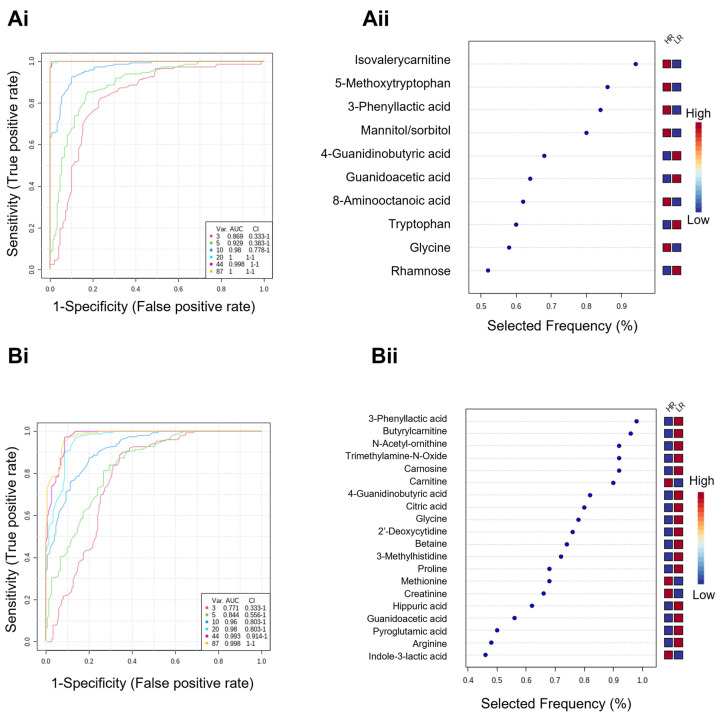
Partial least-squares discriminant analysis (PLS-DA) receiver operating characteristics (ROC) curve and loading plots for significant metabolites discriminating high-responders (HR) and low-responders (LR). Models were generated using datasets comprising (**A**) untreated control and (**B**) drug combination-treated PDE cultures at day 14. (**i**) ROC plots showing area under the curve (AUC) for the models based on different number of features (3, 5, 10, 20, 44 and 87). (**ii**) Frequency percentage plot of the dysregulated metabolites in HR vs. LR in each dataset based on (**A**) 10 and (**B**) 20-features models.

From the binary comparison between HR and LR in the untreated control dataset (Figure 7(Ai,Aii) and Figure S13A), multivariate PLS-DA ROC curve analysis based on a 10 features model (area under the curve (AUC) = 0.98, 95% CI = [0.778–1]) showed a predictive accuracy of 88% (Figure 7(Ai)). The significant features are represented in the selected frequency plots (Figure 7(Aii)). All 10 metabolites had been previously identified as common top features by supervised learning algorithms in this dataset. 4-Guanidinobutyric acid, guanidoacetic acid, tryptophan and rhamnose were up-regulated in LR, whereas the other 6 metabolites were up-regulated in HR (Figure 7(Aii)).

On the other hand, multivariate PLS-DA ROC curve analysis based on a 20-feature model (AUC = 0.98, 95% CI = [0.803–1]) showed a predictive accuracy of 90% (Figure 7(Bi,Bii) and Figure S13B) for the distinction between HR and LR using the combination-treated dataset. It was found that 18 out of 20 metabolites are in common with previously determined common top features by supervised learning algorithms in this dataset. Between both ROC exploring curves, 10-features based on untreated, and 20-features based on drug combination datasets, there were 4 out of 26 unique metabolites in common, namely glycine, guanidoacetic acid, 3-phenyllactic acid, and 4-guanidinobutyric acid.

## 4. Discussion

The prognostic of OvC is still poor, mainly due to the late diagnosis, lack of efficient targeted therapies and high frequency of chemoresistance and tumor relapse, urging the identification of novel and more cancer-specific biomarkers. Our study contributes to the definition of the metabolic footprint underlying chemosensitivity ex vivo, in OvC-PDE cultures. Overall, we established a workflow combining ex vivo models with metabolomics frameworks that paves the way to the identification of systemic prognostic and therapeutic monitoring biomarkers for OvC.

Indirect data, such as with blood-based liquid biopsies, demand especially large patient cohorts due to the low signal-to-noise ratio from the specimen background. Therefore, we hypothesized that metabolomics applied directly to PDE cultures can uncover complex metabolic signatures of treatment response and could be extremely valuable to understand and predict treatment efficacy. We employed an ex vivo model based on the OvC-PDE culture method previously established and characterized by our team. The PDE cultures retain features of the original TME [28] and are suitable for ex vivo drug assays over two weeks, to simulate the cyclic clinical treatment regimens [28,29]. The use of such experimental models to assess drug response and resistance is of utmost interest since TME plays a major role in drug efficacy [24].

As a proof-of-concept of the power of metabolic footprinting to depict metabolic modulation by chemotherapy agents, we performed untargeted metabolomics on two OvC-PDE cultures. Differences between drug-challenged conditions (carboplatin or paclitaxel, as single drugs, or in combination) and untreated controls supported the hypothesis that complex metabolic signatures correlate with OvC-PDE drug response levels and treatment response. Although many efforts have been made towards automated and assisted feature annotation in untargeted analysis, it is still a challenge to translate features into metabolite identities, even with the MS/MS data available, which results in difficult interpretation. Most of the time, few (under 20) metabolites are identified [40,41]. On the other hand, the semi-targeted metabolomic analysis generated a time- and cost-effective metabolic profile. While such an approach still allows for hypothesis generation (as with untargeted metabolomics), metabolites are unambiguously identified [40].

Semi-targeted metabolic footprinting of OvC-PDE cultures derived from eight HGSC allowed the identification of distinct metabolic signatures of HR vs. LR (based on the ex vivo drug combination-induced cell death). We demonstrate that metabolites identified using a high-throughput method (LC-MS) perform well in predicting HR vs. LR. Notably, a panel of metabolites is required for this prediction, which reflects the complexity of the underlying molecular mechanisms. This result could only be captured by the application of multivariate statistical approaches, such as PCA and linear/nonlinear/ensemble machine learning algorithms.

Moreover, our prediction models were based only on eight HGSC PDE cultures, and distinct groups of HR and LR are already observed. Due to the limited amount of tumor samples from which PDE cultures were derived, we used three classification algorithms (PLS-DA, sPLS-DA and RF) to assess the consistency of top metabolic features. We opted for these algorithms since they are feature selection machine learning models widely applied to clinical prediction modeling [42]. Feature selection methods are very useful to deal with large and complex datasets, increasing their interpretability. In general, all models showed a good performance, evaluated by the accuracy, R^2^ and Q^2^ parameters in PLS-DA, all > 0.8; an error rate ≤ 12.5% in sPLS-DA; and an OOB error < 0.4 for RF. Additional LGSC PDE cultures would be required to assess the performance of similar predictive models for LGSC.

We also observed that metabolic profiles hold considerably more information than pathway analysis, which may result from the different scopes of these variables. Top metabolite features identified via machine learning models suggest that more pathways might be affected. Similarly, the classes and related pathways of discriminant metabolites here identified have been previously implicated in OvC pathophysiology and chemoresistance [43,44]. We identified core metabolic routes in cancer in the scope of chemoresistance, namely amino acids, fatty acids, pyrimidine, and TCA cycle metabolic pathways. Thus, the role of amino acids and fatty acids as suppliers of biosynthesis and bioenergetics was reinforced, as well as the metabolic reliance on cysteine and GSH bioavailability as an important mechanism of platinum resistance. In line with our results, distinct metabolic phenotypes related to drug response and resistance were already pointed out in previous studies in 2D [14,45,46,47] and 3D [14] cultures, and in vivo [47]. Dar et al. showed that both established and primary cells isolated from the tumor tissue or ascites of chemo naïve OvC patients displayed distinct metabolic phenotypes, in which a high metabolically active phenotype is correlated with platinum-resistant [45]. Nunes et al. compared 2D and 3D cultures of OvC cell lines and observed that thiol metabolic reliance as a metabolic adjustment upon drugs exposure and accounting for chemoresistance, was similar in both models [14]. Poisson et al. compared the A2780 platinum-sensitive OvC cell line with C200, its platinum-resistant counterpart, and found that pyrimidine metabolism was elevated in resistant cells [46]. In agreement, Nunes et al. showed that the A2780 platinum responsive pattern was positively correlated with the cysteine metabolic reliance [14]. Nucleotides and cysteine metabolism are deeply related to one-carbon (C1) metabolism. Additionally, methionine dependency was observed in resistant OvC cell lines, promoting a higher proliferation and protection against chemotherapeutic agents [48]. The pentose phosphate pathway (PPP) is also very important in redox control because its oxidative branch has the main function of GSH regeneration [49]. The GSH is crucial in OvC platinum resistance and a motor to keep on with the redox balance needed to maintain the metabolic flow [21]. Actually, high PPP activity associated with cisplatin resistance has been reported [50]. Moreover, Hudson et al. reported that platinum-resistant cell lines increase TCA cycle use through glutamine metabolism [51]. Lopes-Coelho et al. reported that in the more platinum-resistant OvC cell line, glutamine is the main precursor of TCA cycle intermediates and glutamate and glycine of GSH molecule [52]. We also found vitamin B6 (pyridoxine) altered, which is considered a central regulator of cisplatin responses in vitro and in vivo [47]. Finally, changes in the tryptophan metabolism were detected and interestingly such alterations have been already shown to promote tumor development and immune suppression [53]. Curiously, tryptophan is also posited as a donor for one-carbon metabolism, being tryptophan-derived, formate is a valuable source for purine nucleotides synthesis [54].

Using statistical methods such as ROC exploratory curves enabled us to turn metabolic signatures into biomarker panels. Biomarker studies using metabolomics for prognosis and correlation with clinical follow-up are scarce in the literature. Chen et al. [55] performed a metabolic profiling analysis on the serum from 36 recurrent and 25 non-recurrent OvC patients. Based on six compounds, namely hypoxanthine, guanidinosuccinic acid, cortisol, lysoPE(22:6) and one of its fragments, and lysoPC(18:2), the classification accuracy using a support vector machine (SVM) model was calculated as 86.9% for non-recurrent and recurrent OvC groups [55]. Sellem et al. [56] used 1H high-resolution magic angle spinning (HRMAS) nuclear magnetic resonance (NMR) spectroscopy for the metabolic characterization of 13 OvC biopsies from responders and two from non-responder patients to the first-line chemotherapy. A PLS-DA model showed a good separation between the groups, although Q^2^ was lower than 0.5. Non-responder patients revealed a higher level of succinate and 3-hydroxybutyrate, whereas the responder patients showed higher levels of glutamate, glutamine, aspartate, and creatine [56]. Our PLS-DA ROC curves show over 88% of accuracy generated from Monte Carlo cross-validation. Some of the metabolites identified in the panel were already described as biomarkers in other studies on OvC and other cancer types. For instance, related to OvC specifically, 3-phenyllactic acid (upregulated in HR) was upregulated in the serum of EOC compared to serum samples from patients with benign ovarian tumors and uterine fibroids [57]; trimethylamine N-oxide (upregulated in LR) was identified in multiple cancers, including OvC [58]; and finally, carnitine (upregulated in LR) levels were increased in serum of OvC patients [59,60], as well as in frozen biopsy tissues from primary and metastatic OvC patients [61]. In fact, alterations in the carnitine system are considered a hallmark of metabolic flexibility [60].

On the other hand, high levels of N-acetyl-ornithine (upregulated in LR) were associated with primary cervical cancer patients and relapse [62]; 3-methylhistidine (upregulated in LR) is reported to be involved in tissue remodeling and repair, inflammation and redox, and protein biosynthesis [63], and it was found elevated in the serum of prostate cancer patients [63] and downregulated in the urine of colorectal cancer patients [64], both compared to healthy controls; 4-guanidinobutyric acid (upregulated in HR) was found to be downregulated in the tissue, urine, and serum of a mouse xenograft model of kidney cancer, compared to the mouse controls [65]; guanidoacetic acid (upregulated in LR) was found to be lower in pancreatic ductal adenocarcinoma tissue compared to the healthy adjacent counterpart [66].

Some other metabolites identified in this study were reported to contribute to chemoresistance within the context of metabolic alterations by the TME. For instance, 2′-deoxycytidine (upregulated in LR) increases in the plasma of cancer patients with poor prognoses; upon chemotherapy, such as cyclophosphamide, methotrexate, and 5-fluorouracil [67]. Iwazaki et al. reported that 2′-deoxycytidine secreted by pancreatic stroma reduces the effect of gemcitabine and other nucleoside analogs on cancer cells, thus contributing to chemotherapy resistance in pancreatic ductal adenocarcinoma [68]. Additionally, we found 5-methoxytryptophan upregulated in HR. This metabolite is secreted by fibroblasts and inhibits cancer cell migration, invasion, tumor growth and metastasis [69,70]. Moreover, other changes in the tryptophan metabolism were detected, such as indole-3-lactic acid (upregulated in HR). Salvador-Coloma et al. identified low levels of this metabolite were correlated with a higher probability of response to neoadjuvant chemotherapy in triple-negative breast cancer [71].

In line with the pathway analysis, once again we identified amino acids (glycine—upregulated in HR) and their degradation products (isovalerylcarnitine—upregulated in HR), which indicates a rapid amino acid catabolism [72]. Hatae et al. showed that blood levels of isovalerylcarnitine tended to increase in the later phase of nivolumab therapy (immunotherapeutic agent) in non-responders compared to responder patients with non-small cell lung cancer [73].

The platform proposed herein has great potential, as discussed above, but still, it has some limitations. The workflow adopted here does not allow capturing the metabolite dynamics in terms of the rate of change of metabolites and flux direction (secretion, uptake, accumulation), even though the experimental platform is compatible with such studies. Because OvC-PDEs are composed of tumor epithelial cells and fibroblasts as the major TME compartment, we cannot distinguish metabolic alterations driven by each compartment. Despite these limitations, we demonstrated that the metabolic changes identified were already pointed out as relevant in metabolic plasticity, drug resistance, and proliferation. Interestingly, despite needing further retrospective studies for approach validation, in the cases where patients received neoadjuvant chemotherapy (OvC1, OvC2 and OvC4), clinical evaluation matched the combination effect evaluated by the log_2_ FC compared to the HSA.

Thus, this platform can be a powerful tool to uncover metabolism-based biomarkers of therapeutic response, patients’ stratification, and adjustments in the clinical management of OvC patients. Ultimately, the panel of markers identified herein should be considered in a follow-up study as systemic prognostic and therapeutic monitoring biomarkers for OvC.

## 5. Conclusions

This study establishes a platform to probe metabolic features of the TME associated with response to treatment. We present a proof-of-concept of the power of this platform by demonstrating that metabolites identified by means of exometabolomic studies in ex vivo patient-derived models, using a high-throughput method (LC-MS), perform well in predicting high or low-responder tissue cultures, identified by the ex vivo drug-induced cell death. Notably, a complex pattern of metabolites propels the prediction and therefore reflects the complexity of the metabolic network underlying molecular mechanisms. Our studies can be a first step toward the identification of biomarkers of chemosensitivity and resistance using patient-derived models. Subsequent prospective studies with a larger patient dataset and control of patient subgroups will allow determining the capability of the tissue cultures to predict clinical outcomes and validate a biomarker panel for clinical use. In fact, most of the biomarkers here identified were reported to be involved in OvC pathophysiology, and response to therapy, as well as in other cancer types. Further characterization of these distinct profiles can provide mechanistic insights into the metabolic crosstalk between cancer and TME cells, in this case, epithelial tumor cells and fibroblasts. Biomarkers identified in the TME exometabolome can potentially be released into the bloodstream and be easily accessible for patient stratification as a serum biomarker, improving clinical management [74,75]. Due to the universal nature of metabolites, such platforms based on exometabolomics can potentially be applied to different in vitro or ex vivo disease models. Understanding diseases on their molecular level will support the development of precision medicine and hence lead to better prognostic and therapeutic monitoring in the clinics. Earlier detection of therapeutic resistance can improve patient selection towards appropriate treatment regimens, either for targeted therapies currently in the guidelines or for their earlier incorporation in clinical trials with new therapeutic agents. Moreover, identifying metabolic signatures that are intrinsically different across patient-derived samples can serve not only as a tool to predict therapy response but also as a drug discovery platform, specifically for finding metabolism-targeting therapies for resistant OvC tumors [46].

## Data Availability

The datasets used and/or analyzed during the current study are available from the corresponding author on reasonable request.

## Supplementary Materials

The following supporting information can be downloaded at https://www.mdpi.com/article/10.3390/cancers14184460/s1. File S1: Supplementary Figures, comprising: Figure S1: Sample-specific response to combination (C+P) and single drugs cyclic drug exposure in serous OvC patient cohort; Figure S2: Metabolomics data preprocessing; Figure S3: Metabolomics data pretreatment; Figure S4: Untargeted metabolic footprints distinct treatment groups of 2 HGSC OvC-PDE samples using RPLC in the positive mode; Figure S5: Cross-validation and 100 permutation tests for PLS-DA classification using HILIC(+), HILIC(−), and RPLC(−) datasets; Figure S6: Spectra of top discriminating features uncovered by untargeted metabolomics in datasets HILIC(+), HILIC(−) and RPLC(−); Figure S7: Spectra of the metabolites present in the basal medium (DMEM) used to culture OvC-PDE; Figure S8: Semi-targeted analysis all samples (OvC1–9); Figure S9: Multivariate data analysis using dataset comprising single-agents treated samples at day 14; Figure S10: Supervised analysis using dataset comprising untreated control samples at day 14; Figure S11: Multivariate data analysis using dataset comprising combination (C+P) treated PDE cultures dataset at day 14; Figure S12: Untreated and drug combination challenged PDE cultures metabolic traits determine ex vivo drug response; Figure S13: PLS-DA ROC curve analysis using dataset comprising untreated control PDE cultures and combination (C+P) treated samples at day 14; File S2: Untargeted metabolomics metadata, data matrixes—HILIC(+), HILIC(−), RPLC(+) and RPLC(−)—and feature annotation table; File S3: Semi-targeted metabolomics data by annotation level (1, 2a, 2b, 3 and unknown); File S4: Multivariate data analysis using principal component regression (PCR) and table of top case-intrinsic and drug-induced discriminating metabolites and pathways.

## References

[1] Matulonis U.A., Sood A.K., Fallowfield L., Howitt B.E., Sehouli J., Karlan B.Y. (2016). Ovarian Cancer. Nat. Rev. Dis. Primers.

[2] Rojas V., Hirshfield K.M., Ganesan S., Rodriguez-Rodriguez L. (2016). Molecular Characterization of Epithelial Ovarian Cancer: Implications for Diagnosis and Treatment. Int. J. Mol. Sci..

[3] Plotti F., Terranova C., Guzzo F., Nardone C.D.C., Luvero D., Bartolone M., Dionisi C., Benvenuto D., Fabris S., Ciccozzi M. (2021). Role of BRCA Mutation and He4 in Predicting Chemotherapy Response in Ovarian Cancer: A Retrospective Pilot Study. Biomedicines.

[4] Ledermann J.A., Raja F.A., Fotopoulou C., Gonzalez-Martin A., Colombo N., Sessa C. (2013). Newly Diagnosed and Relapsed Epithelial Ovarian Carcinoma: ESMO Clinical Practice Guidelines for Diagnosis, Treatment and Follow-Up. Ann. Oncol..

[5] Radu M.R., Prădatu A., Duică F., Micu R., Creţoiu S.M., Suciu N., Creţoiu D., Varlas V.N., Rădoi V.E. (2021). Ovarian Cancer: Biomarkers and Targeted Therapy. Biomedicines.

[6] Von Strandmann E.P., Reinartz S., Wager U., Müller R. (2017). Tumor—Host Cell Interactions in Ovarian Cancer: Pathways to Therapy Failure. Trends Cancer.

[7] Luo Z., Wang Q., Lau W.B., Lau B., Xu L., Zhao L., Yang H., Feng M., Xuan Y., Yang Y. (2016). Tumor Microenvironment: The Culprit for Ovarian Cancer Metastasis?. Cancer Lett..

[8] Santo V.E., Rebelo S.P., Estrada M.F., Alves P.M., Boghaert E., Brito C. (2017). Drug Screening in 3D in Vitro Tumor Models: Overcoming Current Pitfalls of Efficacy Read-Outs. Biotechnol. J..

[9] Puchades-Carrasco L., Pineda- Lucena A. (2017). Metabolomics Applications in Precision Medicine: An Oncological Perspective. Curr. Top. Med. Chem..

[10] Wishart D.S. (2016). Emerging Applications of Metabolomics in Drug Discovery and Precision Medicine. Nat. Rev. Drug Discov..

[11] Ward P.S., Thompson C.B. (2012). Metabolic Reprogramming: A Cancer Hallmark Even Warburg Did Not Anticipate. Cancer Cell.

[12] Zaal E.A., Berkers C.R. (2018). The Influence of Metabolism on Drug Response in Cancer. Front. Oncol..

[13] Nunes S.C., Ramos C., Santos I., Mendes C., Silva F., Vicente J.B., Pereira S.A., Félix A., Gonçalves L.G., Serpa J. (2021). Cysteine Boosts Fitness Under Hypoxia-Mimicked Conditions in Ovarian Cancer by Metabolic Reprogramming. Front. Cell Dev. Biol..

[14] Nunes S.C., Ramos C., Lopes-Coelho F., Sequeira C.O., Silva F., Gouveia-Fernandes S., Rodrigues A., Guimarães A., Silveira M., Abreu S. (2018). Cysteine Allows Ovarian Cancer Cells to Adapt to Hypoxia and to Escape from Carboplatin Cytotoxicity. Sci. Rep..

[15] Nunes S.C., Lopes-Coelho F., Gouveia-Fernandes S., Ramos C., Pereira S.A., Serpa J. (2018). Cysteine Boosters the Evolutionary Adaptation to CoCl2 Mimicked Hypoxia Conditions, Favouring Carboplatin Resistance in Ovarian Cancer. BMC Evol. Biol..

[16] Lau A.N., Heiden M.G. (2020). Vander Metabolism in the Tumor Microenvironment. Annu. Rev. Cancer Biol..

[17] Elia I., Haigis M.C. (2021). Metabolites and the Tumour Microenvironment: From Cellular Mechanisms to Systemic Metabolism. Nat. Metab..

[18] Thuwajit C., Ferraresi A., Titone R., Thuwajit P., Isidoro C. (2018). The Metabolic Cross-Talk between Epithelial Cancer Cells and Stromal Fibroblasts in Ovarian Cancer Progression: Autophagy Plays a Role. Med. Res. Rev..

[19] Dasari S., Fang Y., Mitra A.K. (2018). Cancer Associated Fibroblasts: Naughty Neighbors That Drive Ovarian Cancer Progression. Cancers.

[20] Cheteh E.H., Augsten M., Rundqvist H., Bianchi J., Sarne V., Egevad L., Bykov V.J.N., Östman A., Wiman K.G. (2017). Human Cancer-Associated Fibroblasts Enhance Glutathione Levels and Antagonize Drug-Induced Prostate Cancer Cell Death. Cell Death Dis..

[21] Nunes S.C., Serpa J. (2018). Glutathione in Ovarian Cancer: A Double-Edged Sword. Int. J. Mol. Sci..

[22] Chen Y., Guo W., Fan J., Chen Y., Zhang X., Chen X., Luo P. (2017). The Applications of Liquid Biopsy in Resistance Surveillance of Anaplastic Lymphoma Kinase Inhibitor. Cancer Manag. Res..

[23] Snow A., Chen D., Lang J.E. (2019). The Current Status of the Clinical Utility of Liquid Biopsies in Cancer. Expert Rev. Mol. Diagn..

[24] Sun Y. (2016). Tumor Microenvironment and Cancer Therapy Resistance. Cancer Lett..

[25] Rodenhizer D., Dean T., D’Arcangelo E., McGuigan A.P. (2018). The Current Landscape of 3D In Vitro Tumor Models: What Cancer Hallmarks Are Accessible for Drug Discovery?. Adv. Healthc. Mater..

[26] Muir A., Danai L.V., vander Heiden M.G. (2018). Microenvironmental Regulation of Cancer Cell Metabolism: Implications for Experimental Design and Translational Studies. Dis. Models Mech..

[27] Powley I.R., Patel M., Miles G., Pringle H., Howells L., Thomas A., Kettleborough C., Bryans J., Hammonds T., MacFarlane M. (2020). Patient-Derived Explants (PDEs) as a Powerful Preclinical Platform for Anti-Cancer Drug and Biomarker Discovery. Br. J. Cancer.

[28] Abreu S., Silva F., Mendes R., Mendes T.F., Teixeira M., Santo V.E., Boghaert E.R., Félix A., Brito C. (2020). Patient-Derived Ovarian Cancer Explants: Preserved Viability and Histopathological Features in Long-Term Agitation-Based Cultures. Sci. Rep..

[29] Cox M.C., Mendes R., Silva F., Mendes T.F., Zelaya-Lazo A., Halwachs K., Purkal J.J., Isidro I.A., Félix A., Boghaert E.R. (2021). Application of LDH Assay for Therapeutic Efficacy Evaluation of Ex Vivo Tumor Models. Sci. Rep..

[30] Bioconductor—XCMS. https://bioconductor.org/packages/release/bioc/html/xcms.html.

[31] Chong J., Soufan O., Li C., Caraus I., Li S., Bourque G., Wishart D.S., Xia J. (2018). MetaboAnalyst 4.0: Towards More Transparent and Integrative Metabolomics Analysis. Nucleic Acids Res..

[32] Wishart D.S., Tzur D., Knox C., Eisner R., Guo A.C., Young N., Cheng D., Jewell K., Arndt D., Sawhney S. (2007). HMDB: The Human Metabolome Database. Nucleic Acids Res..

[33] Guijas C., Montenegro-Burke J.R., Domingo-Almenara X., Palermo A., Warth B., Hermann G., Koellensperger G., Huan T., Uritboonthai W., Aisporna A.E. (2018). METLIN: A Technology Platform for Identifying Knowns and Unknowns. Anal. Chem..

[34] Wang M., Carver J.J., Phelan V.V., Sanchez L.M., Garg N., Peng Y., Nguyen D.D., Watrous J., Kapono C.A., Luzzatto-Knaan T. (2016). Sharing and Community Curation of Mass Spectrometry Data with GNPS. Nat. Biotechnol..

[35] Schrimpe-Rutledge A.C., Codreanu S.G., Sherrod S.D., Mclean J.A. (2016). Untargeted Metabolomics Strategies—Challenges and Emerging Directions. J. Am. Soc. Mass Spectrom..

[36] Doneanu C.E., Chen W., Mazzeo J.R., Corporation W. (2011). UPLC/MS Monitoring of Water-Soluble Vitamin Bs in Cell Culture Media in Minutes. Waters Appl. Notes.

[37] Pang Z., Chong J., Zhou G., De Lima Morais D.A., Chang L., Barrette M., Gauthier C., Jacques P.É., Li S., Xia J. (2021). MetaboAnalyst 5.0: Narrowing the Gap between Raw Spectra and Functional Insights. Nucleic Acids Res..

[38] Foucquier J., Guedj M. (2015). Analysis of Drug Combinations: Current Methodological Landscape. Pharmacol. Res. Perspect..

[39] Wang M.C., Li S. (2013). ROC Analysis for Multiple Markers with Tree-Based Classification. Lifetime Data Anal..

[40] Liu X., Locasale J.W. (2017). Metabolomics: A Primer. Trends Biochem. Sci..

[41] Zamboni N., Saghatelian A., Patti G.J. (2015). Defining the Metabolome: Size, Flux, and Regulation. Mol. Cell.

[42] Jamshidi A., Pelletier J.P., Martel-Pelletier J. (2019). Machine-Learning-Based Patient-Specific Prediction Models for Knee Osteoarthritis. Nat. Rev. Rheumatol..

[43] Lyssiotis C.A., Kimmelman A.C. (2017). Metabolic Interactions in the Tumor Microenvironment. Trends Cell Biol..

[44] Ferraresi A., Girone C., Esposito A., Vidoni C., Vallino L., Secomandi E., Dhanasekaran D.N., Isidoro C. (2020). How Autophagy Shapes the Tumor Microenvironment in Ovarian Cancer. Front. Oncol..

[45] Dar S., Chhina J., Mert I., Chitale D., Buekers T., Kaur H., Giri S., Munkarah A., Rattan R. (2017). Bioenergetic Adaptations in Chemoresistant Ovarian Cancer Cells. Sci. Rep..

[46] Poisson L.M., Munkarah A., Madi H., Datta I., Hensley-Alford S., Tebbe C., Buekers T., Giri S., Rattan R. (2015). A Metabolomic Approach to Identifying Platinum Resistance in Ovarian Cancer. J. Ovarian Res..

[47] Galluzzi L., Vitale I., Senovilla L., Olaussen K.A., Pinna G., Eisenberg T., Goubar A., Martins I., Michels J., Kratassiouk G. (2012). Prognostic Impact of Vitamin B6 Metabolism in Lung Cancer. Cell Rep..

[48] Wang Z., Yip L.Y., Lee J.H.J., Wu Z., Chew H.Y., Chong P.K.W., Teo C.C., Ang H.Y.K., Peh K.L.E., Yuan J. (2019). Methionine Is a Metabolic Dependency of Tumor-Initiating Cells. Nat. Med..

[49] Gough N.R. (2015). PPP to the Rescue. Sci. Signal..

[50] Giacomini I., Ragazzi E., Pasut G. (2020). The Pentose Phosphate Pathway and Its Involvement in Cisplatin Resistance. Int. J. Mol. Sci..

[51] Hudson C.D., Savadelis A., Nagaraj A.B., Joseph P., Avril S., DiFeo A., Avril N. (2016). Altered Glutamine Metabolism in Platinum Resistant Ovarian Cancer. Oncotarget.

[52] Lopes-Coelho F., Gouveia-Fernandes S., Gonçalves L.G., Nunes C., Faustino I., Silva F., Félix A., Pereira S.A., Serpa J. (2016). HNF1β Drives Glutathione (GSH) Synthesis Underlying Intrinsic Carboplatin Resistance of Ovarian Clear Cell Carcinoma (OCCC). Tumor Biol..

[53] Xu Y., Zhang H., Sun Q., Geng R., Yuan F., Liu B., Chen Q. (2021). Immunomodulatory Effects of Tryptophan Metabolism in the Glioma Tumor Microenvironment. Front. Immunol..

[54] Newman A.C., Falcone M., Huerta Uribe A., Zhang T., Athineos D., Pietzke M., Vazquez A., Blyth K., Maddocks O.D.K. (2021). Immune-Regulated IDO1-Dependent Tryptophan Metabolism Is Source of One-Carbon Units for Pancreatic Cancer and Stellate Cells. Mol. Cell.

[55] Chen J., Zhang Y., Zhang X., Cao R., Chen S., Huang Q., Lu X., Wan X., Wu X., Xu C. (2011). Application of L-EDA in Metabonomics Data Handling: Global Metabolite Profiling and Potential Biomarker Discovery of Epithelial Ovarian Cancer Prognosis. Metabolomics.

[56] Namer I.J., ben Sellem D., Elbayed K., Neuville A., Moussallieh F.M., Lang-Averous G., Piotto M., Bellocq J.P. (2011). Metabolomic Characterization of Ovarian Epithelial Carcinomas by HRMAS-NMR Spectroscopy. J. Oncol..

[57] Ke C., Hou Y., Zhang H., Fan L., Ge T., Guo B., Zhang F., Yang K., Wang J., Lou G. (2015). Large-Scale Profiling of Metabolic Dysregulation in Ovarian Cancer. Int. J. Cancer.

[58] Xu R., Wang Q.Q., Li L. (2015). A Genome-Wide Systems Analysis Reveals Strong Link between Colorectal Cancer and Trimethylamine N-Oxide (TMAO), a Gut Microbial Metabolite of Dietary Meat and Fat. BMC Genom..

[59] Console L., Scalise M., Mazza T., Pochini L., Galluccio M., Giangregorio N., Tonazzi A., Indiveri C. (2020). Carnitine Traffic in Cells. Link with Cancer. Front. Cell Dev. Biol..

[60] Saorin A., Gregorio E.D., Miolo G., Ste A., Corona G. (2020). Emerging Role of Metabolomics in Ovarian Cancer Diagnosis. Metabolites.

[61] Fong M.Y., McDunn J., Kakar S.S. (2011). Identification of Metabolites in the Normal Ovary and Their Transformation in Primary and Metastatic Ovarian Cancer. PLoS ONE.

[62] Zhou H., Li Q., Wang T., Liang H., Wang Y., Duan Y., Song M., Wang Y., Jin H. (2020). Exploring Metabolomics Biomarkers for Evaluating the Effectiveness of Concurrent Radiochemotherapy for Cervical Cancers. Transl. Cancer Res..

[63] Huang J., Mondul A.M., Weinstein S.J., Karoly E.D., Sampson J.N., Albanes D. (2017). Prospective Serum Metabolomic Profile of Prostate Cancer by Size and Extent of Primary Tumor. Oncotarget.

[64] Deng Y., Yao H., Chen W., Wei H., Li X., Zhang F., Gao S., Man H., Chen J., Tao X. (2020). Profiling of Polar Urine Metabolite Extracts from Chinese Colorectal Cancer Patients to Screen for Potential Diagnostic and Adverse-Effect Biomarkers. J. Cancer.

[65] Ganti S., Taylor S.L., Aboud O.A., Yang J., Evans C., Osier M.V., Alexander D.C., Kim K., Weiss R.H. (2012). Kidney Tumor Biomarkers Revealed by Simultaneous Multiple Matrix Metabolomics Analysis. Cancer Res..

[66] Kamphorst J.J., Nofal M., Commisso C., Hackett S.R., Lu W., Grabocka E., vander Heiden M.G., Miller G., Drebin J.A., Bar-Sagi D. (2015). Human Pancreatic Cancer Tumors Are Nutrient Poor and Tumor Cells Actively Scavenge Extracellular Protein. Cancer Res..

[67] Iwazaki A., Imai K., Nakanishi K., Yoshioka M. (2010). Changes in 2′-Deoxycytidine Levels in Various Tissues of Tumor-Bearing Mice. Oncol. Lett..

[68] Dalin S., Sullivan M.R., Lau A.N., Grauman-Boss B., Mueller H.S., Kreidl E., Fenoglio S., Luengo A., Lees J.A., vander Heiden M.G. (2019). Deoxycytidine Release from Pancreatic Stellate Cells Promotes Gemcitabine Resistance. Cancer Res..

[69] Wu K.K., Cheng H.H., Chang T.C. (2014). 5-Methoxyindole Metabolites of L-Tryptophan: Control of COX-2 Expression, Inflammation and Tumorigenesis. J. Biomed. Sci..

[70] Wu K.K. (2021). Cytoguardin: A Tryptophan Metabolite against Cancer Growth and Metastasis. Int. J. Mol. Sci..

[71] Salvador-Coloma C., Santaballa A., Sanmartín E., Calvo D., García A., Hervás D., Cordón L., Quintas G., Ripoll F., Panadero J. (2020). Immunosuppressive Profiles in Liquid Biopsy at Diagnosis Predict Response to Neoadjuvant Chemotherapy in Triple-Negative Breast Cancer. Eur. J. Cancer.

[72] Halama A., Guerrouahen B.S., Pasquier J., Diboun I., Karoly E.D., Suhre K., Rafii A. (2015). Metabolic Signatures Differentiate Ovarian from Colon Cancer Cell Lines. J. Transl. Med..

[73] Hatae R., Chamoto K., Kim Y.H., Sonomura K., Taneishi K., Kawaguchi S., Yoshida H., Ozasa H., Sakamori Y., Akrami M. (2020). Combination of Host Immune Metabolic Biomarkers for the PD-1 Blockade Cancer Immunotherapy. JCI Insight.

[74] Kell D.B., Brown M., Davey H.M., Dunn W.B., Spasic I., Oliver S.G. (2005). Metabolic Footprinting and Systems Biology: The Medium Is the Message. Nat. Rev. Microbiol..

[75] Knott M.E., Manzi M., Zabalegui N., Salazar M.O., Puricelli L.I., Monge M.E. (2018). Metabolic Footprinting of a Clear Cell Renal Cell Carcinoma in Vitro Model for Human Kidney Cancer Detection. J. Proteome Res..

