# Supplementary material for: Exploring Metabolic Signatures of Ex Vivo Tumor Tissue Cultures for Prediction of Chemosensitivity in Ovarian Cancer
Source: Cancers (Basel). 2022 Sep 14;14(18):4460. doi: 10.3390/cancers14184460 (PMC9496731; doi:10.3390/cancers14184460)
Supplement: Supplementary file 1 [file cancers-14-04460-s001.zip › Supplementary File S1_SI figures.pdf]

Supplementary File S1

Exploring Metabolic Signatures of Ovarian Cancer Ex Vivo Models to Predict Drug Efficacy

Rita Mendes [1,2], Gonçalo Graça [3], Fernanda Silva [4], Ana C. L. Guerreiro [1,2], Patrícia Gomes-Alves [1,2], Jacinta Serpa [4,5], Erwin R. Boghaert [6], Ana Félix [4,5], Catarina Brito [1,2], Inês A. Isidro [1,2,*]

[1] iBET, Instituto de Biologia Experimental e Tecnológica, Apartado 12, 2781-901 Oeiras, Portugal

[2] Instituto de Tecnologia Química e Biológica António Xavier, Universidade Nova de Lisboa, Av. da República, 2780-157 Oeiras, Portugal

[3] Department of Metabolism, Digestion and Reproduction, Imperial College London, London SW7 2AZ, UK

[4] iNOVA4Health, NOVA Medical School/Faculdade de Ciências Médicas, Universidade NOVA de Lisboa, Campo dos Mártires da Pátria, 130, 1169-056 Lisboa, Portugal

[5] Molecular Pathobiology Unit, Instituto Português de Oncologia de Lisboa Francisco Gentil (IPOLFG), Rua Prof. Lima Basto, 1099-023 Lisboa, Portugal

[6] AbbVie, 1 North Waukegan Road, North Chicago, IL 60064-6098, USA

[7] Pathology Department, Instituto Português de Oncologia de Lisboa Francisco Gentil (IPOLFG), Rua Prof. Lima Basto, 1099-023 Lisboa, Portugal

* Correspondence: iaisidro@ibet.pt

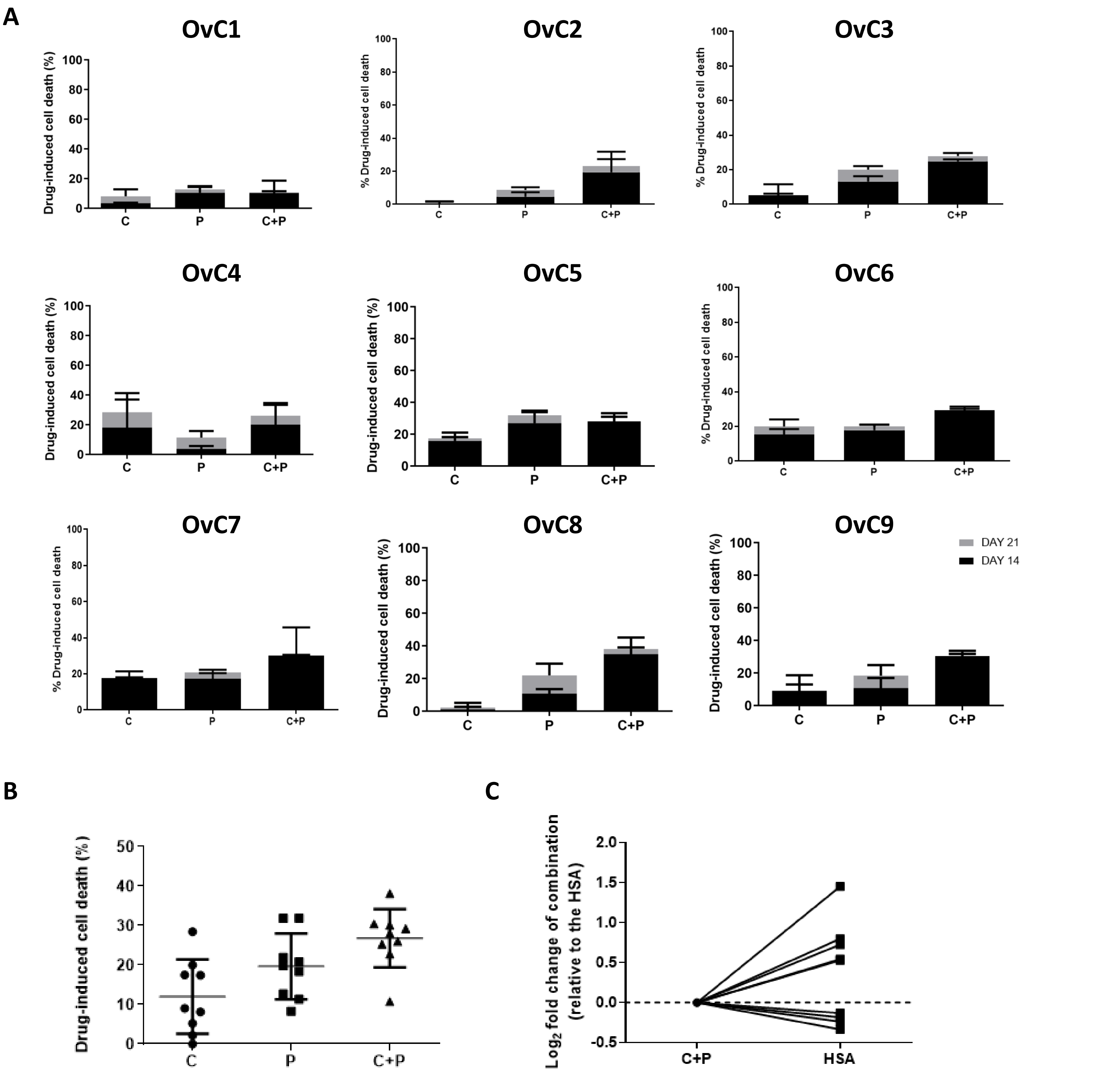

Supplementary Figure S1: Sample-specific response to combination (C+P) and single drugs cyclic drug exposure in serous OvC patient cohort. (A) Drug-induced cell death evaluation of OvC-PDE using LDH assay after 2 drug cycles. Data is presented as mean ± SD of three technical replicates per OvC-PDE case. **(B)** Drug-induced cell death of OvC-PDE along two drug cycles, evaluated by the LDH assay. **(C)** Log2 fold change of the combination drug-induced cell death relative to the drug-induced cell death of the highest single agent (HSA). (N=9, C: carboplatin, P: paclitaxel; LGSC: low-grade serous carcinoma).

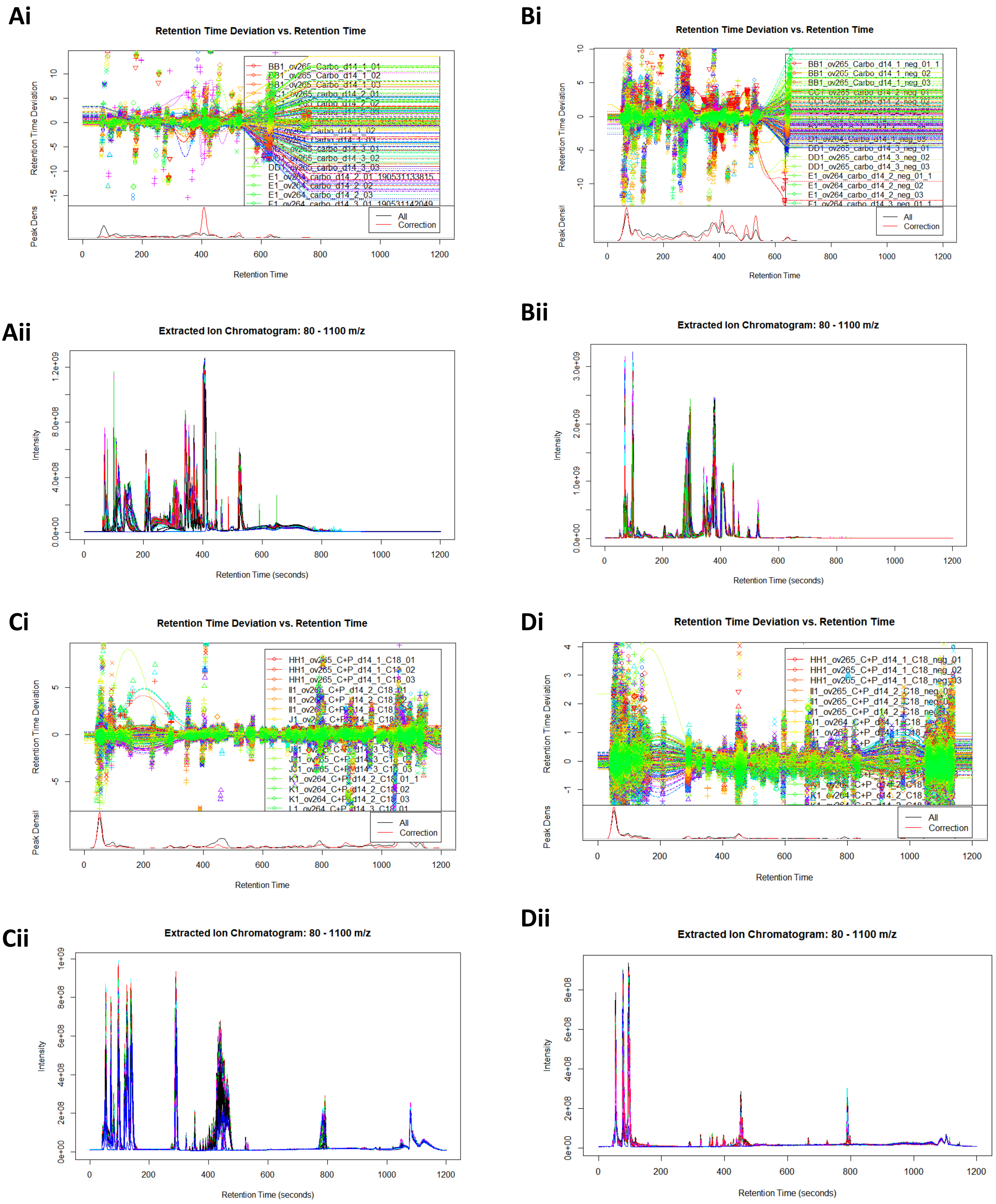

Supplementary Figure S2: Metabolomics data preprocessing: Peak alignment (i) and extracted ion chromatogram (EIC) (ii) using XCMS software applied in the four untargeted metabolomics datasets. (A) HILIC(+); **(B)** HILIC(-); **(C)** RPLC(+); and **(D)** RPLC(-). (HILIC: hydrophilic interaction liquid chromatography; RPLC: reversed phase liquid chromatography;(+):positive mode; (-):negative mode)

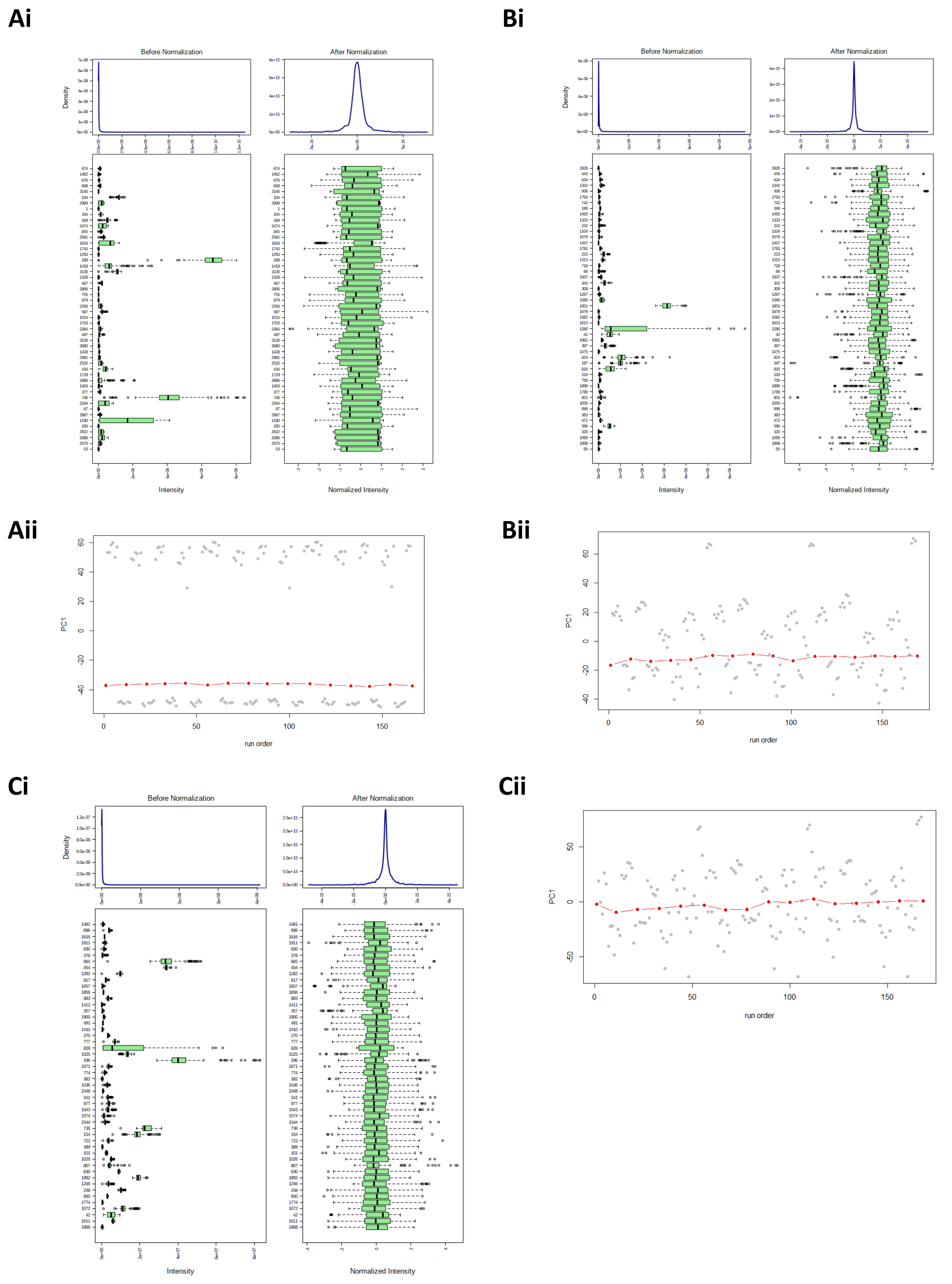

Supplementary Figure S3: Metabolomics data pretreatment in untargeted metabolomics datasets. (i) Normalization, including scaling and transformation, of peak intensities across features and (ii) stability of QCs over injection run. QCs in red; samples in grey. **(A)** HILIC(+); **(B)** HILIC(-); **(C)** RPLC(+); and **(D)** RPLC(-). (HILIC: hydrophilic interaction liquid chromatography; RPLC: reversed phase liquid chromatography; (+):positive mode; (-):negative mode, QC: quality control samples).

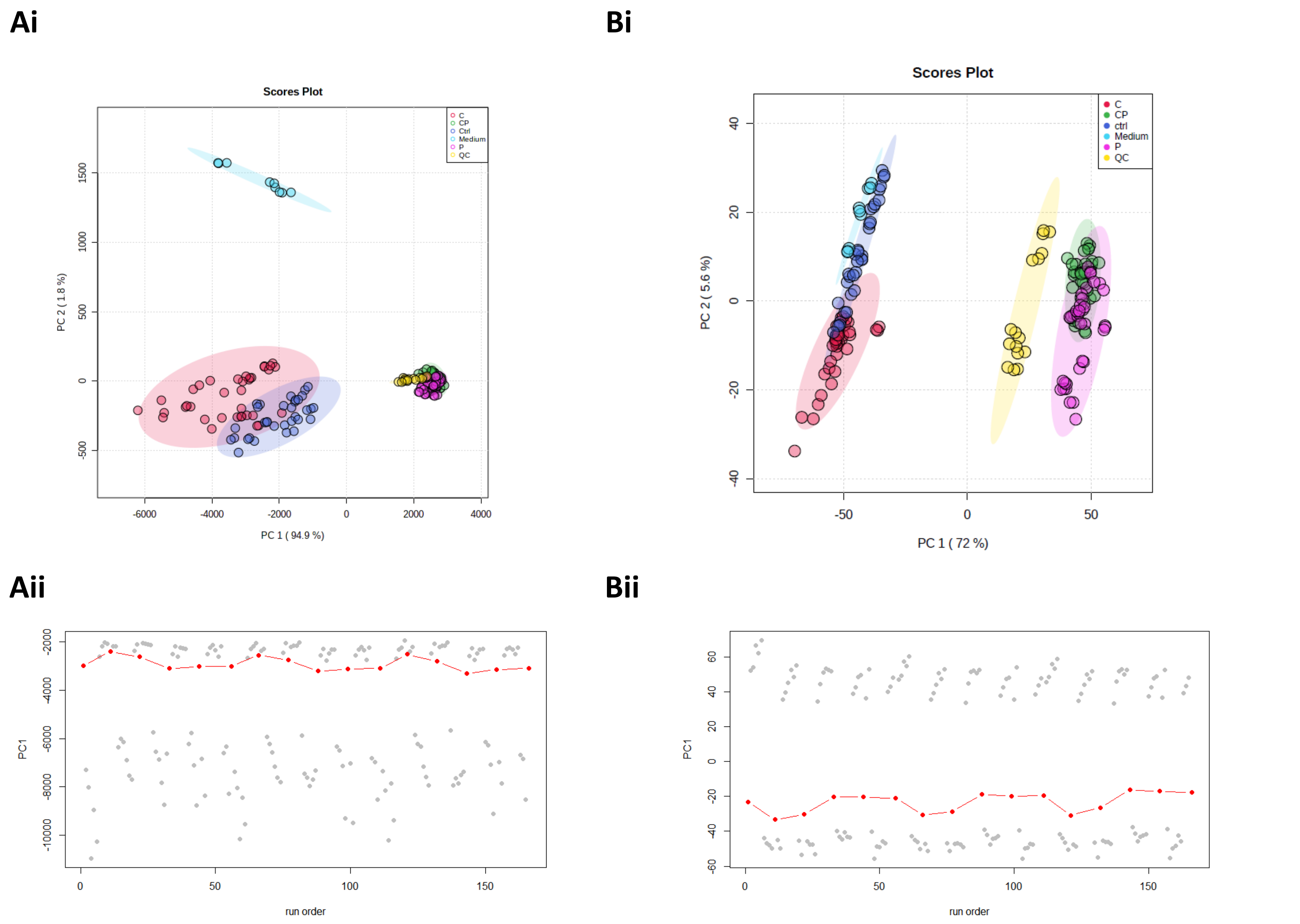

Supplementary Figure S4: Untargeted metabolic footprints distinct treatment groups of 2 HGSC OvC-PDE samples using RPLC in the positive mode. Multivariate data analysis performed using **(i)** principal component analysis (PCA) **(A)** when samples were normalized by the median or **(B)** normalized as other columns/modes and **(ii)** stability of QCs over injection run (ii: QCs in red; samples in grey) (C: carboplatin, P: paclitaxel; ctrl: untreated control; Medium: culture media blanks; QC: quality control sample; RPLC: reversed phase liquid chromatography)

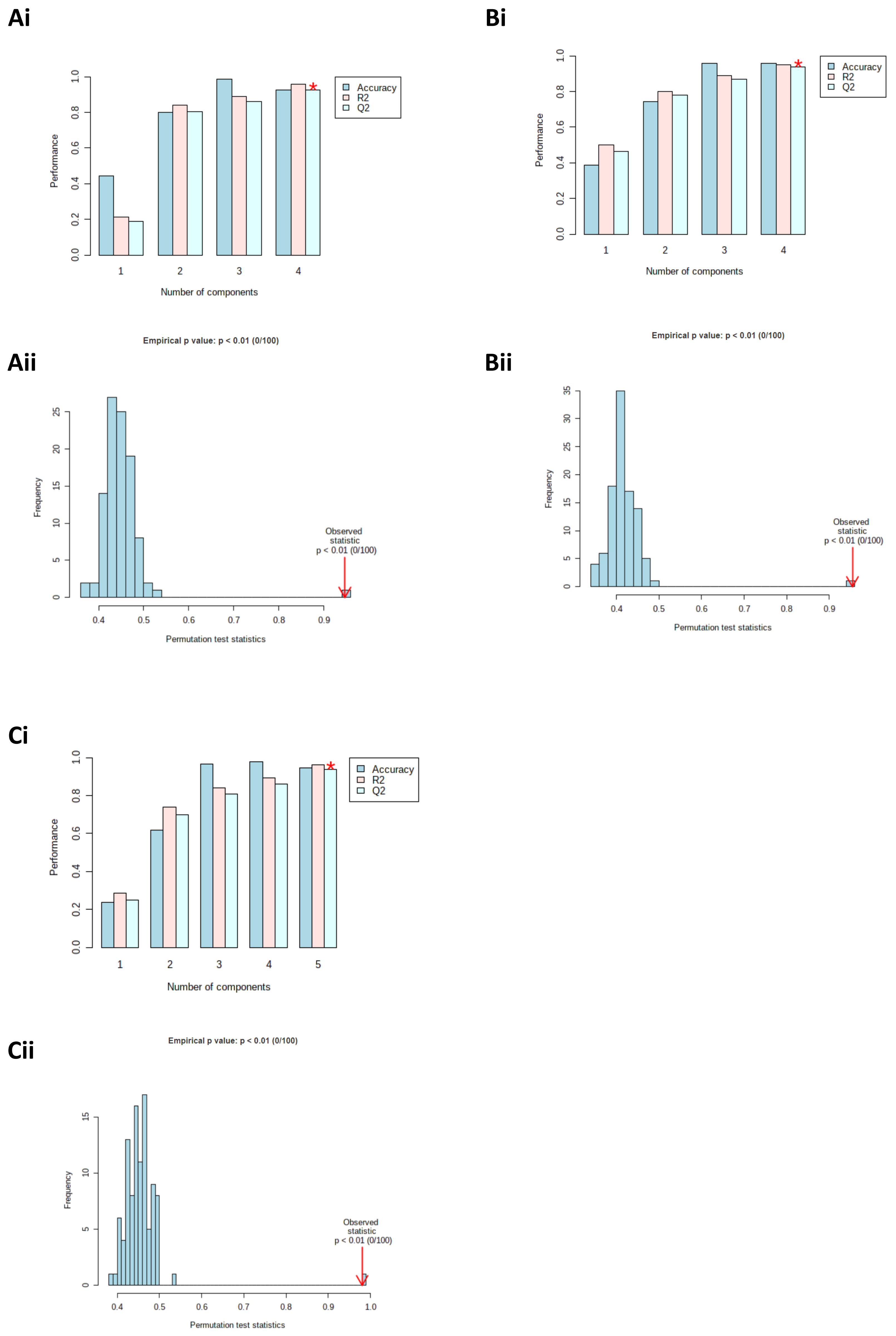

Supplementary Figure S5: Cross-validation (i) and 100 permutation tests (ii) for PLS-DA classification using (A) HILIC(+); **(B)** HILIC(-); and **(C)** RPLC(-) datasets. ((HILIC: hydrophilic interaction liquid chromatography; RPLC: reversed phase liquid chromatography; (+):positive mode; (-):negative mode)

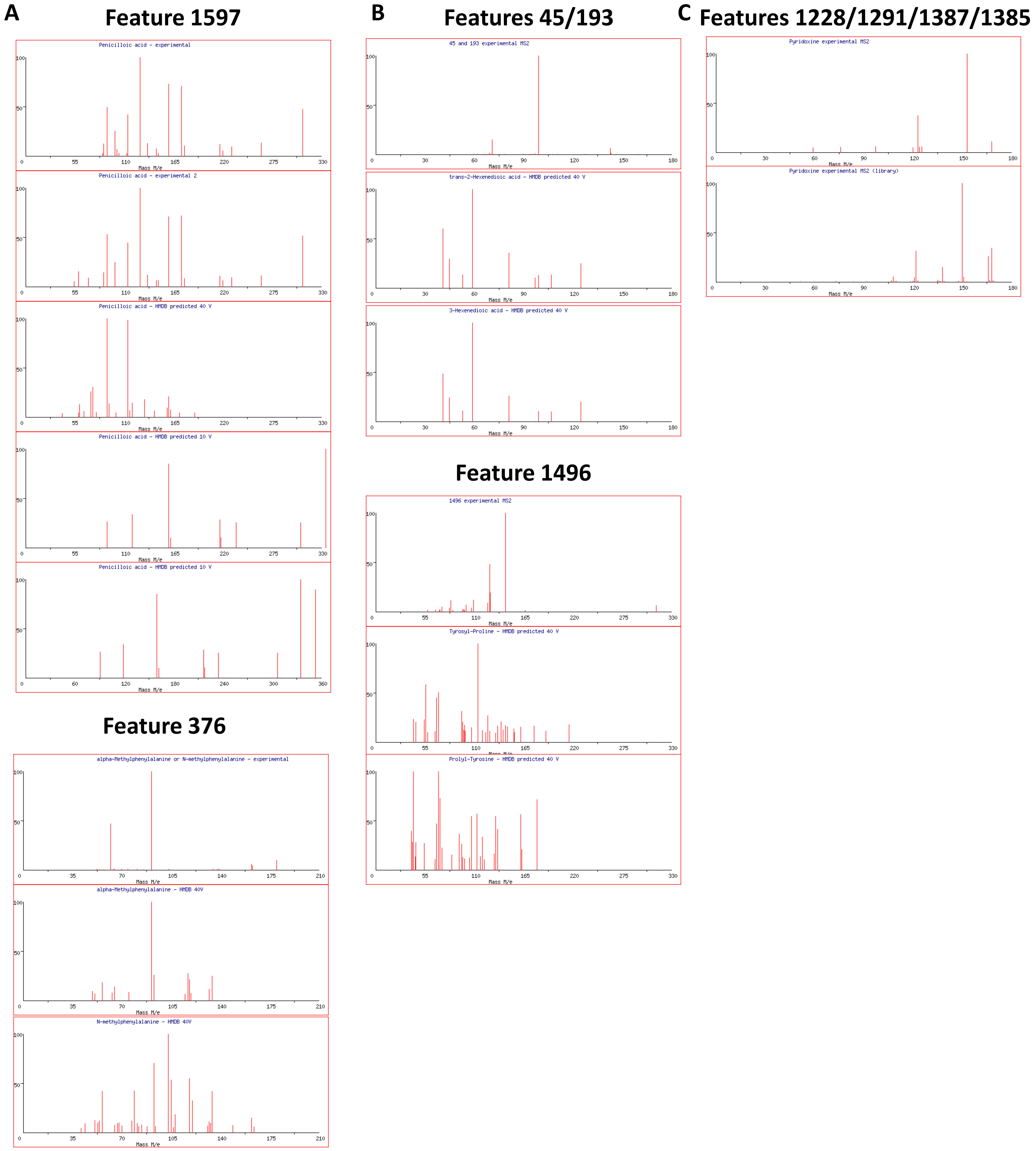

Supplementary Figure S6: Spectra of top discriminating features uncovered by untargeted metabolomics in datasets (A) HILIC(+), (B) HILIC(-) and (C) RPLC(-). (upper: experimental MS2 and lower: MS2 spectra from library) (HILIC: hydrophilic interaction liquid chromatography; RPLC: reversed phase liquid chromatography; (+):positive mode; (-):negative mode)

L-Glutamine

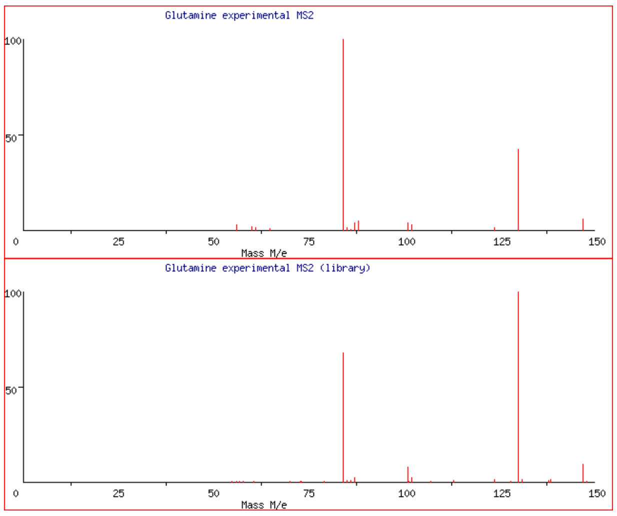

L-Valine

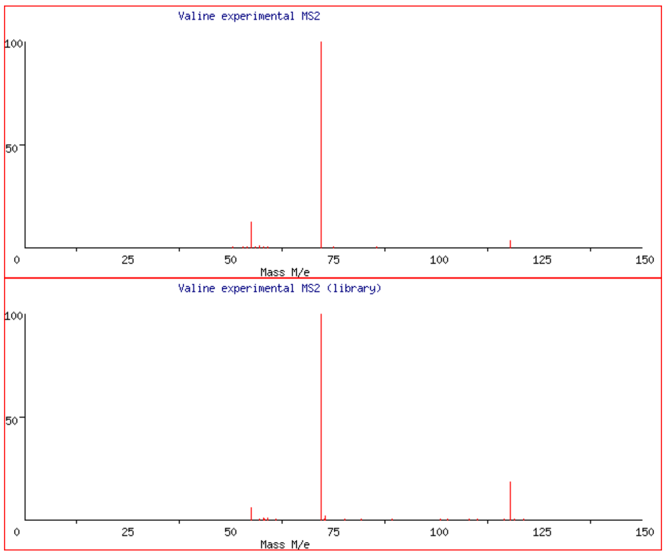

L-Methionine

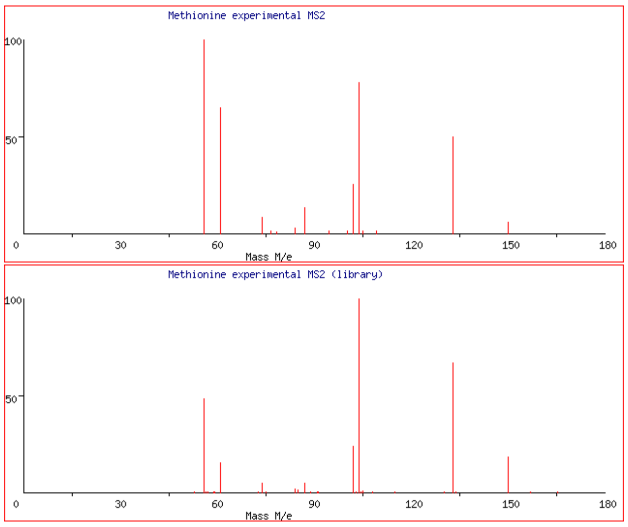

Niacinamide

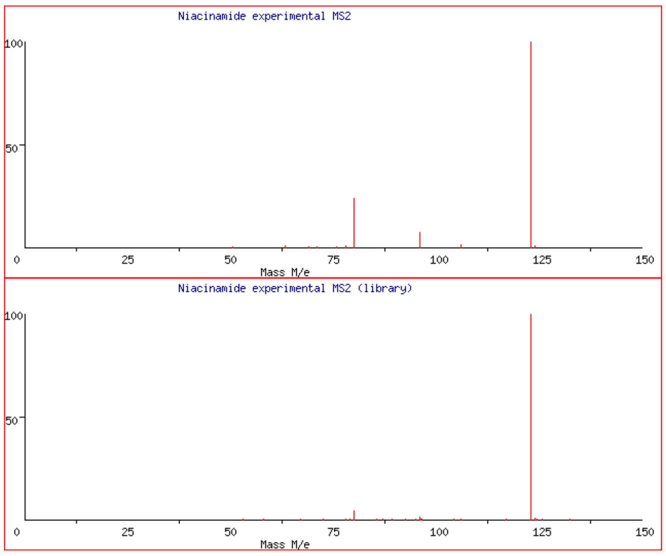

L-Isoleucine/L-Leucine

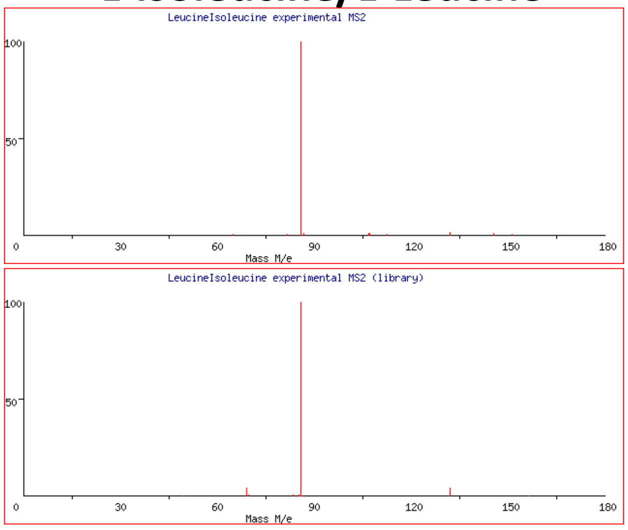

Hexose

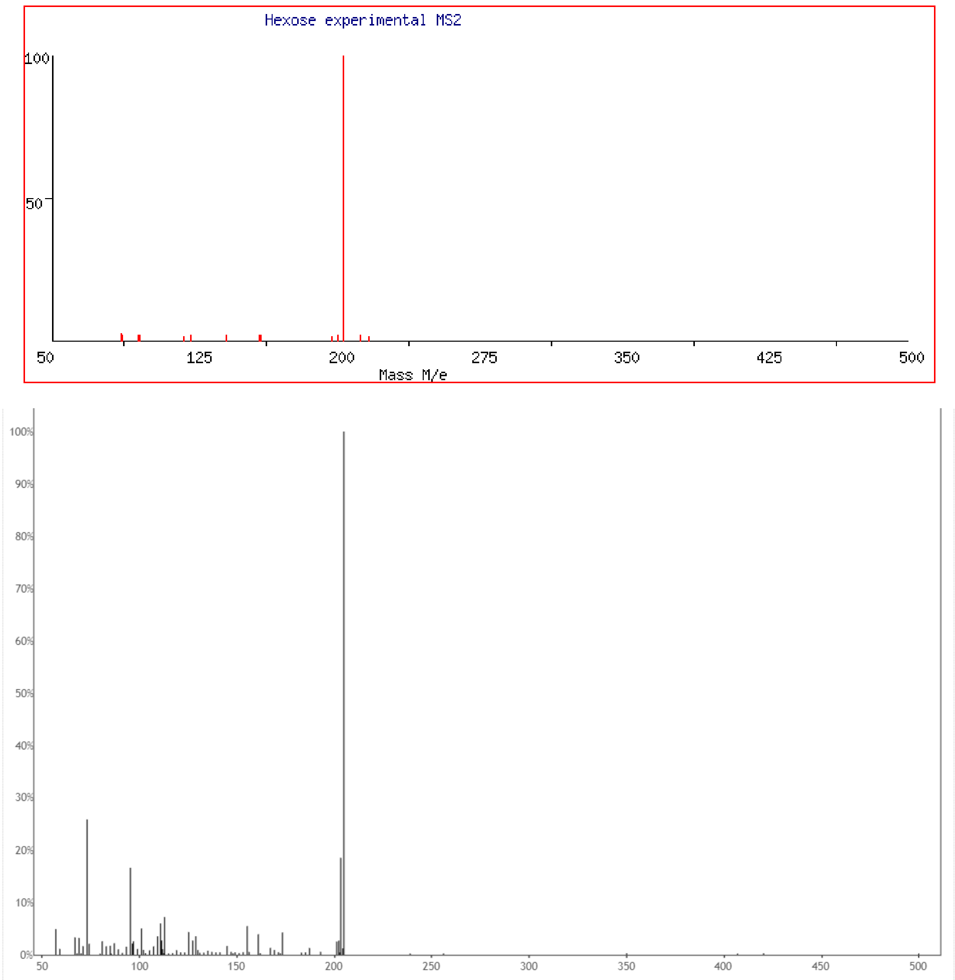

L-Phenylalanine

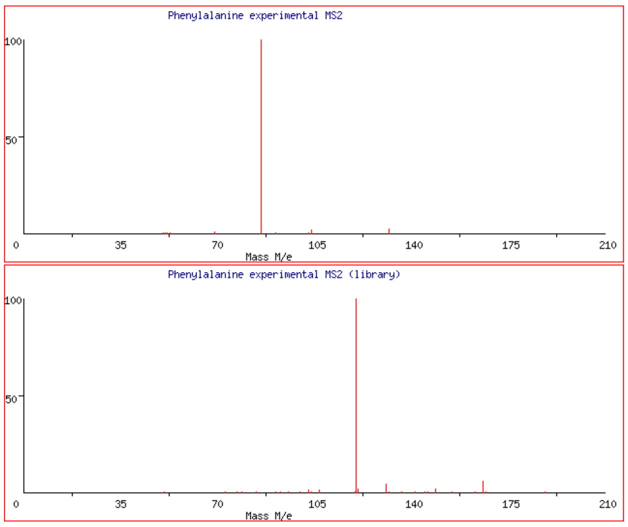

Supplementary Figure S7: Spectra of the metabolites present in the basal medium (DMEM) used to culture OvC-PDE.(upper: experimental MS2 and lower: MS2 spectra from library)

A

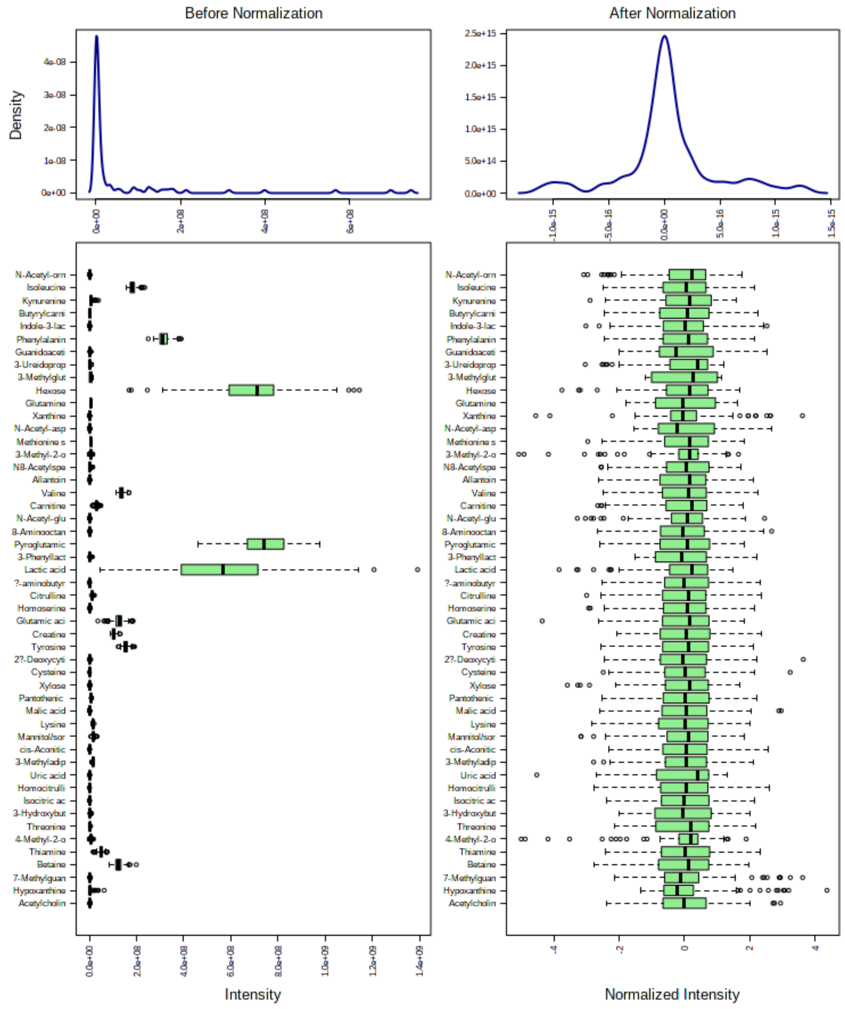

B

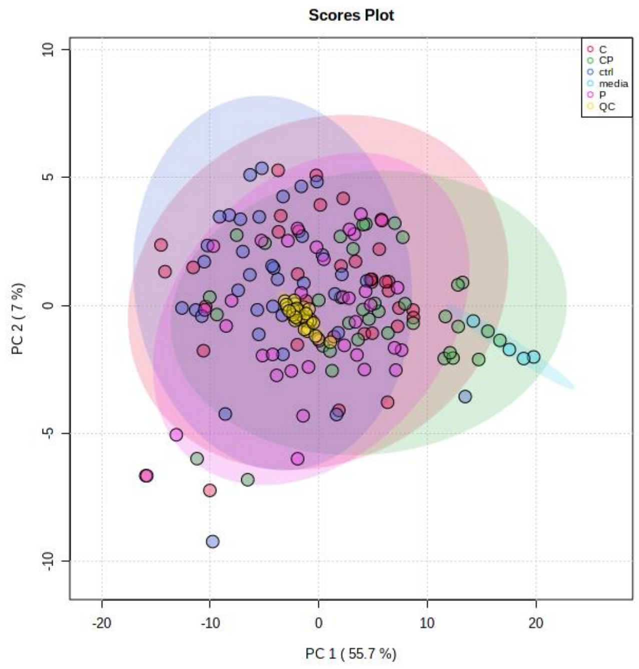

Supplementary Figure S8: Semi-targeted analysis all samples (OvC1-9). (A) Normalization, including scaling and transformation, of peak intensities across features. **(B)** Multivariate data analysis performed using principal component analysis. (the dataset used comprised all samples: untreated controls and treated conditions at day 14 and 21) (C: carboplatin, P: paclitaxel; Ctrl: untreated control; Medium: culture media blanks; QC: quality control sample)

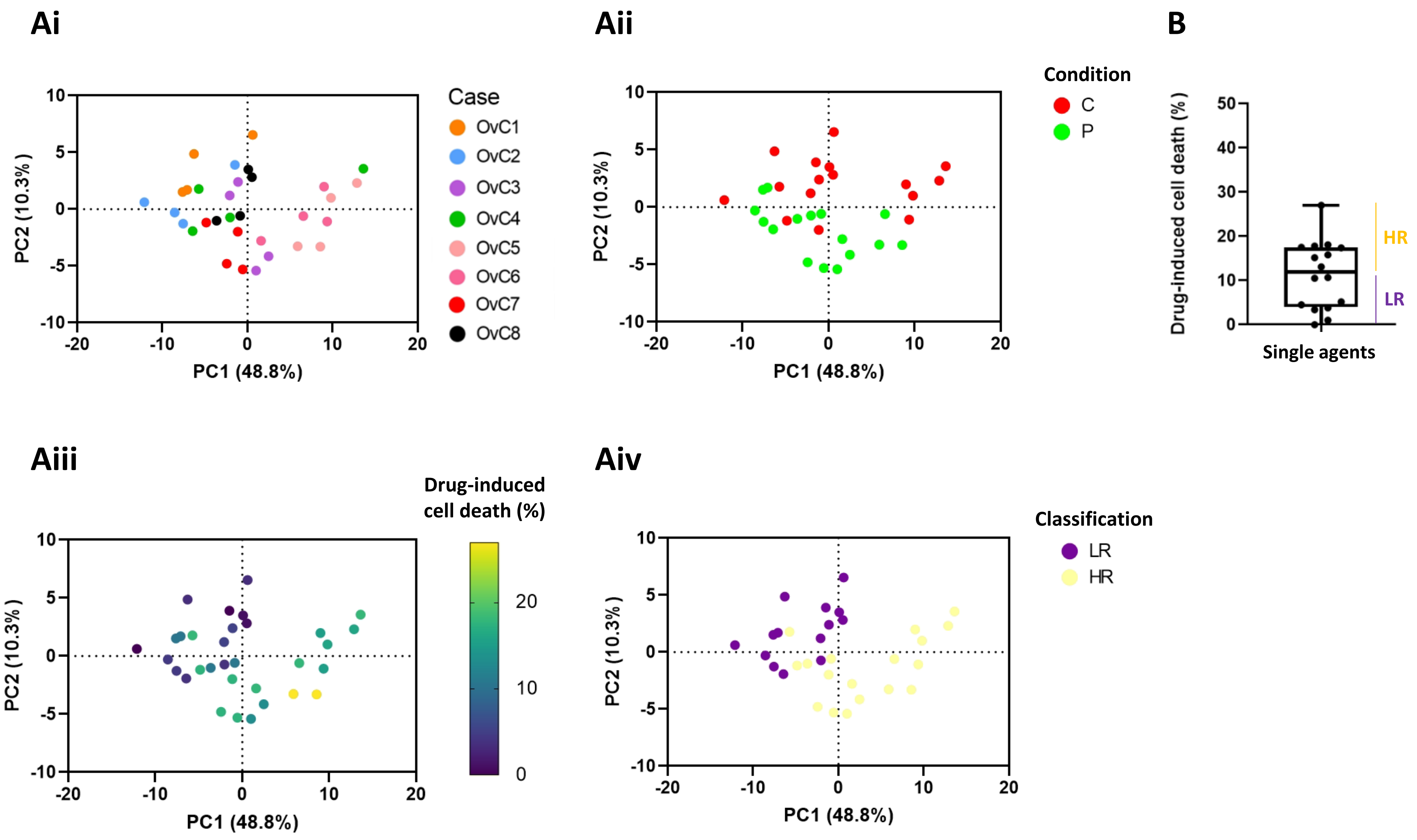

Supplementary Figure S9: Multivariate data analysis using dataset comprising single-agents treated samples at day 14. Unsupervised analysis using **(A)** PCA by **(Ai)** OvC-PDE case; **(ii)** single-agent treatment (C vs P); drug response trends by the **(iii)** drug-induced cell death after one drug cycle (day 14) and **(iv)** based on high-responder (HR) and low-responder (LR) classification. **(B)** Cut-off between HR and LR based on the median drug-induced cell death for single drug-treated conditions. (C: carboplatin; P: paclitaxel; HR: high-responder; LR: low-responder; PCA: principal component analysis)

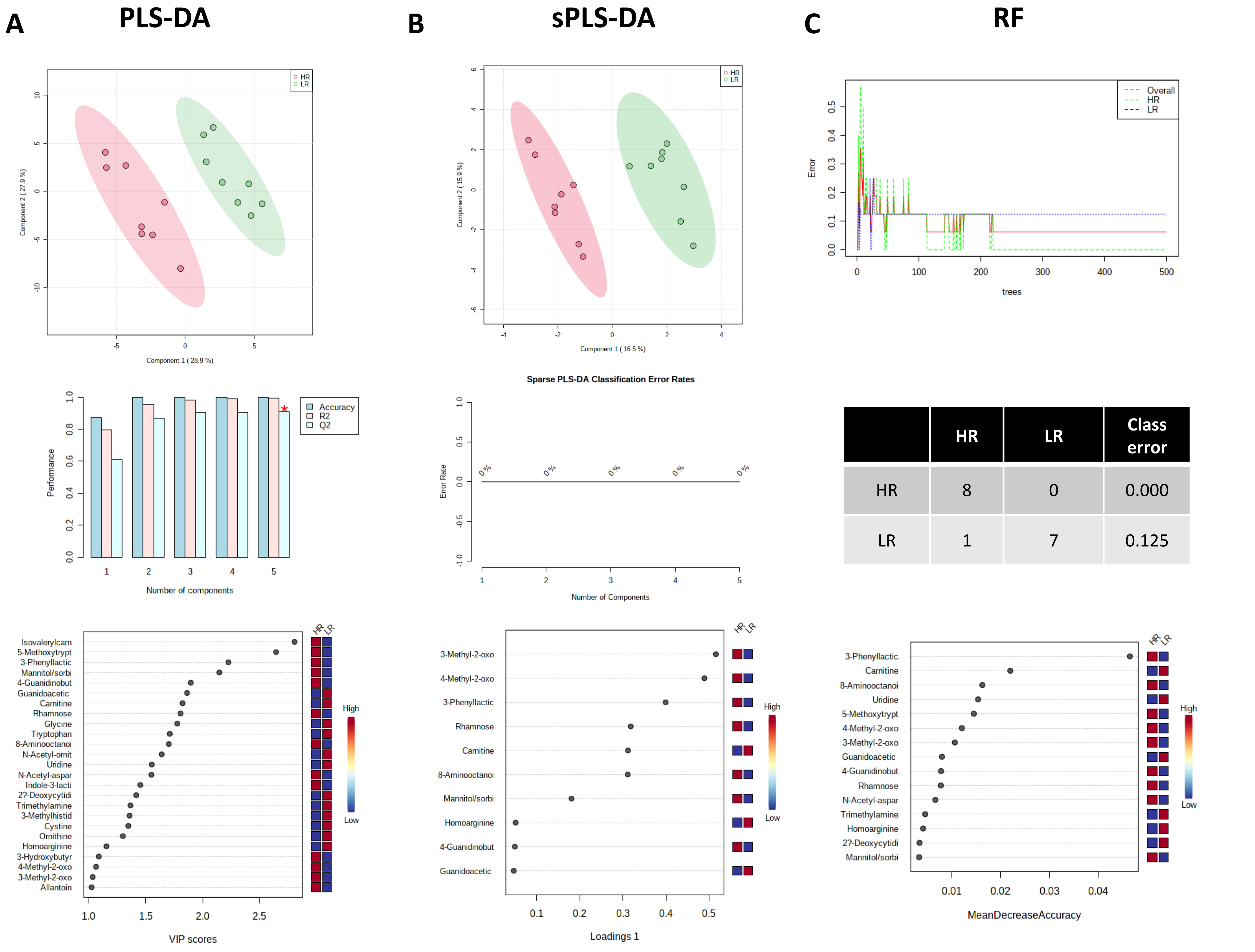

	HR	LR	Class error
HR	8	0	0.000
LR	1	7	0.125

Supplementary Figure S10: Supervised analysis using dataset comprising untreated control PDE cultures at day 14. (A) PLS-DA; **(B)** sPLS-DA and **(C)** RF models, with respective plots (upper panel), performance (middle panel) and top discriminating features (lower panel) separating HR vs LR. (HR: high-responder; LR: low-responder; PLS-DA: partial least-squares discriminant analysis; sPLS-DA: sparse partial least-squares discriminant analysis; RF: random forests)

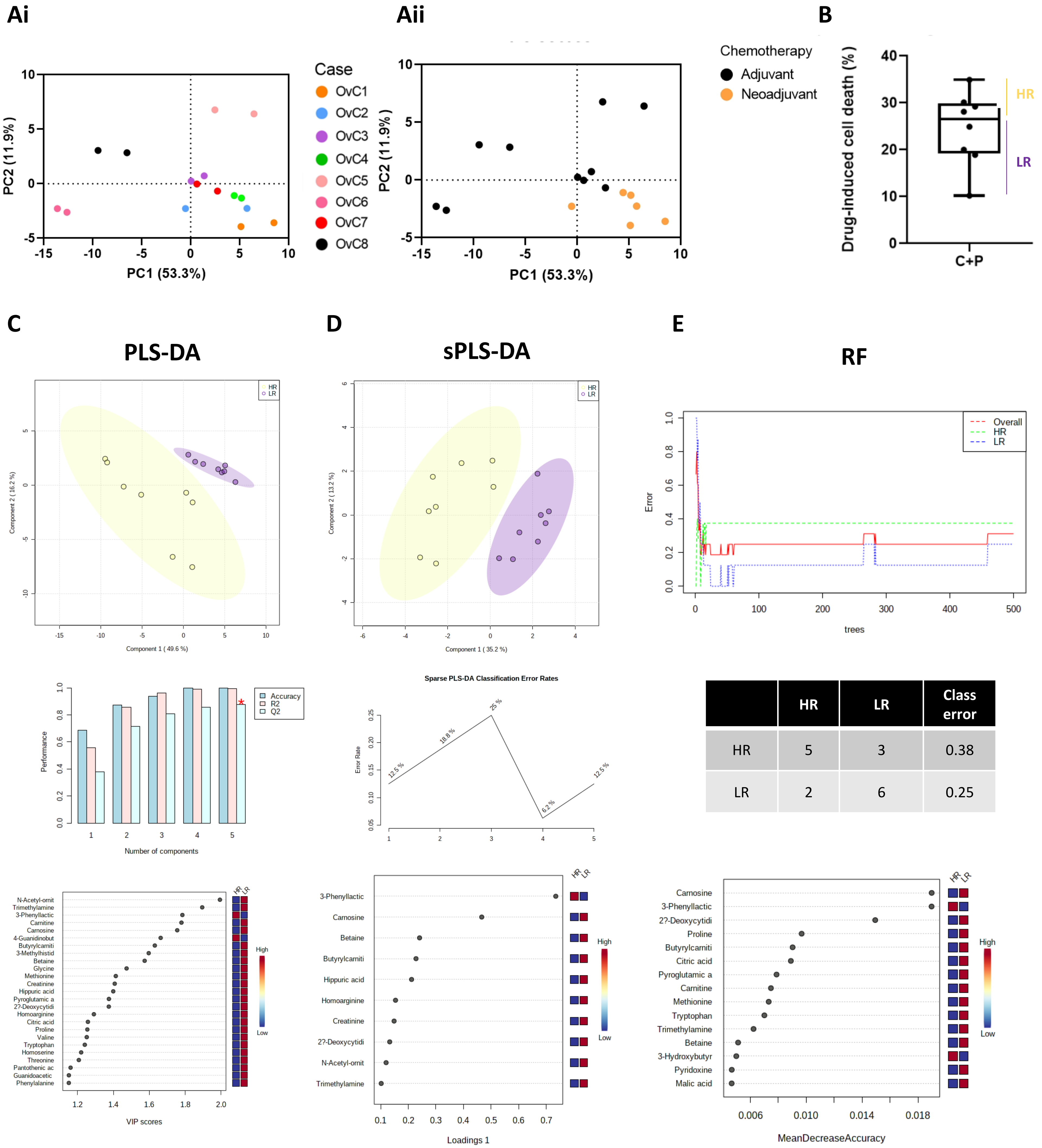

Supplementary Figure S11: Multivariate data analysis using dataset comprising combination (C+P) treated PDE cultures dataset at day 14. Unsupervised analysis using **(A)** PCA by **(Ai)** OvC-PDE case and **(ii)** chemotherapy regimen (adjuvant vs neoadjuvant) **(B)** Cut-off between high-responders (HR) and low-responders (LR) based on the % drug-induced cell death median. Supervised analysis using **(C)** PLS-DA; **(D)** sPLS-DA and **(E)** RF models, with respective plots (upper panel), performance (middle panel) and top discriminating features (lower panel) separating HR vs LR. (C: carboplatin; P: paclitaxel; HR: high-responder; LR: low-responder; PLS-DA: partial least-squares discriminant analysis; sPLS-DA: sparse partial least-squares discriminant analysis; RF: random forests)

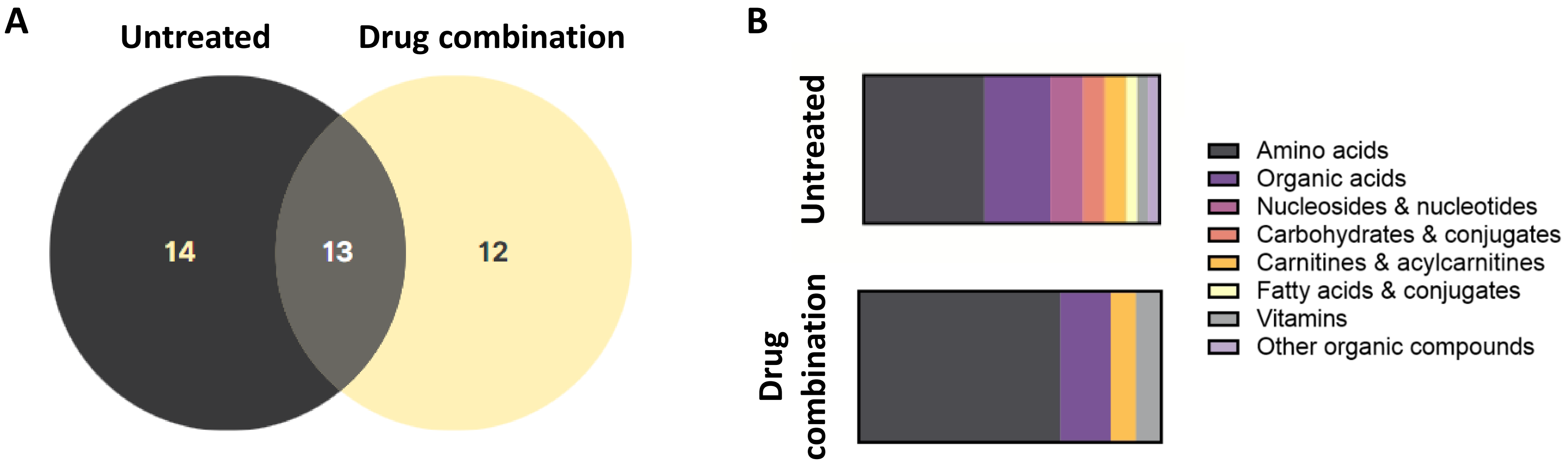

Supplementary Figure S12: Untreated and drug combination challenged PDE cultures metabolic traits determine ex vivo drug response. (A) Top discriminating metabolic features for drug response (high-responders, HR versus low-responders, LR) in untreated and combination-treated PDE datasets and **(B)** respective metabolite classes.

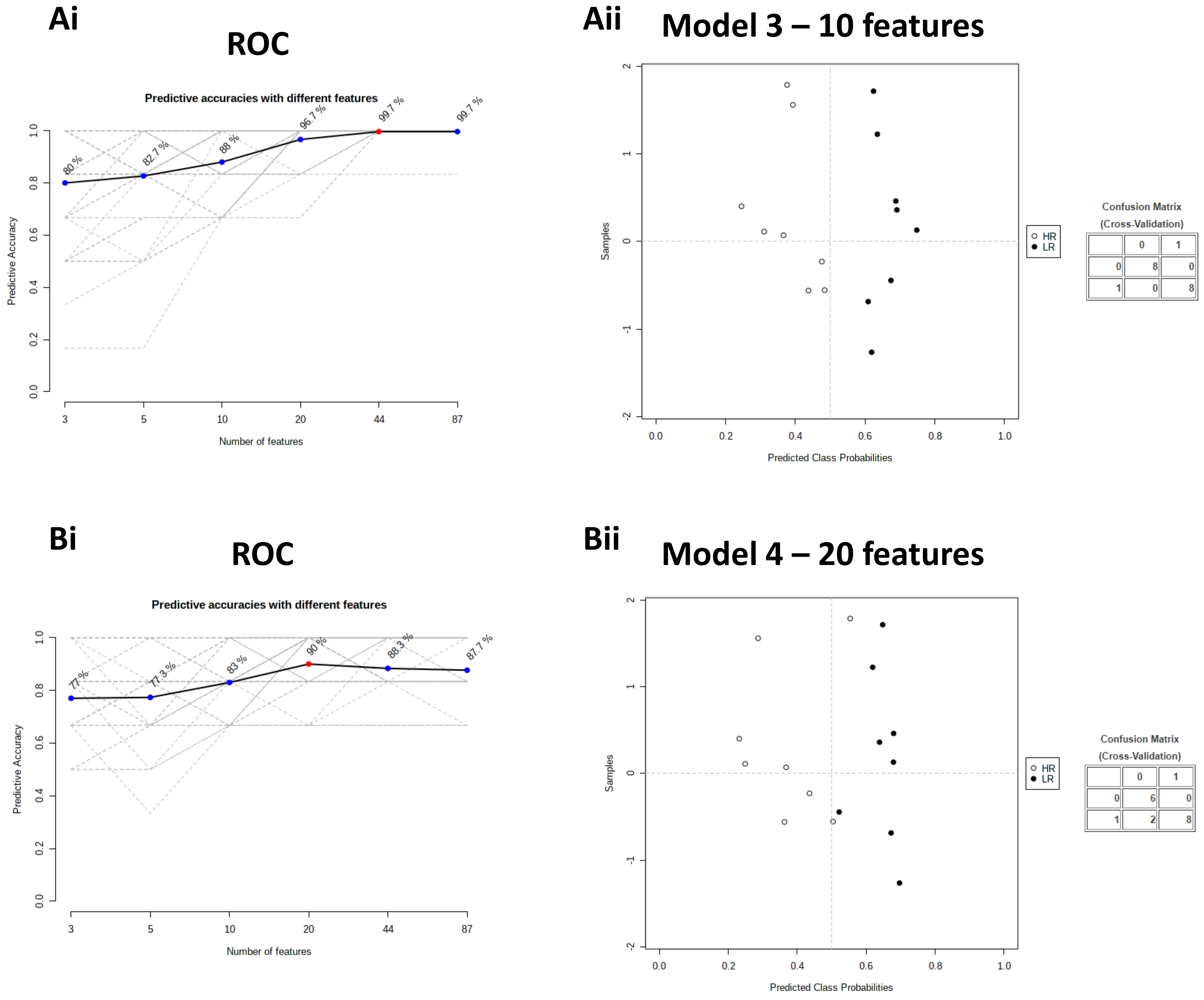

Supplementary Figure S13: PLS-DA ROC curve analysis using dataset comprising (A) untreated control PDE cultures and (B) combination (C+P) treated samples at day 14. (i) Accuracy using different number of features. **(ii)** No class error was observed for a model based on **(Aii)** 10 features (or more features) or **(Bii)** 20 features (optimal accuracy - red dot. (C: carboplatin; P: paclitaxel; HR: high-responder; LR: low-responder; PLS-DA: partial least-squares discriminant analysis; ROC: receiver operating curve)
